# Suppressing mitochondrial inner membrane protein (IMMT) inhibits the proliferation of breast cancer cells through mitochondrial remodeling and metabolic regulation

**DOI:** 10.1038/s41598-024-63427-8

**Published:** 2024-06-04

**Authors:** Li Liu, Qingqing Zhao, Daigang Xiong, Dan Li, Jie Du, Yunfei Huang, Yan Yang, Rui Chen

**Affiliations:** 1 Clinical Medical College, Zunyi Medical University, Zunyi, China; 2 Department of Laboratory Medicine, Affiliated Hospital of ZunYi Medical University, Zunyi, China; 3 School of Laboratory Medicine, Zunyi Medical University, Zunyi, China; 4 Department of General Surgery, Affiliated Hospital of Zunyi Medical University, Zunyi, China; 5 Department of Thyroid and Breast Surgery, Affiliated Hospital of Zunyi Medical University, Zunyi, China

**Keywords:** Mitochondrial inner membrane protein (IMMT), Breast cancer, Mitochondria remodeling, Metabolic reprogramming, Prognostic marker, Cancer, Breast cancer, Cancer metabolism

## Abstract

Metabolic reprogramming is widely recognized as a hallmark of malignant tumors, and the targeting of metabolism has emerged as an appealing approach for cancer treatment. Mitochondria, as pivotal organelles, play a crucial role in the metabolic regulation of tumor cells, and their morphological and functional alterations are intricately linked to the biological characteristics of tumors. As a key regulatory subunit of mitochondria, mitochondrial inner membrane protein (IMMT), plays a vital role in degenerative diseases, but its role in tumor is almost unknown. The objective of this research was to investigate the roles that IMMT play in the development and progression of breast cancer (BC), as well as to elucidate the underlying biological mechanisms that drive these effects. In this study, it was confirmed that the expression of IMMT in BC tissues was significantly higher than that in normal tissues. The analysis of The Cancer Genome Atlas (TCGA) database revealed that IMMT can serve as an independent prognostic factor for BC patients. Additionally, verification in clinical specimens of BC demonstrated a positive association between high IMMT expression and larger tumor size (> 2 cm), Ki-67 expression (> 15%), and HER-2 status. Furthermore, in vitro experiments have substantiated that the suppression of IMMT expression resulted in a reduction in cell proliferation and alterations in mitochondrial cristae, concomitant with the liberation of cytochrome c, but it did not elicit mitochondrial apoptosis. Through Gene Set Enrichment Analysis (GSEA) analysis, we have predicted the associated metabolic genes and discovered that IMMT potentially modulates the advancement of BC through its interaction with 16 metabolic-related genes, and the changes in glycolysis related pathways have been validated in BC cell lines after IMMT inhibition. Consequently, this investigation furnishes compelling evidence supporting the classification of IMMT as prognostic marker in BC, and underscoring its prospective utility as a novel target for metabolic therapy.

## Introduction

In terms of malignancies in women, breast cancer (BC) is the most common and one of the leading causes of cancer death worldwide^1^. The mortality of BC patients has been significantly reduced by radical surgery, radiotherapy, chemotherapy, endocrine therapy, and targeted therapy within the past decade, but many patients still experience recurrence or metastasis, eventually leading to death^2^. The utilization of molecular typing, specifically with regards to the estrogen and progesterone receptors (ER and PR), human epidermal growth factor receptor 2 (HER-2), and other markers, has significantly enhanced the precision of treatment for BC. However, the biological diversity of this disease presents a formidable obstacle in the development of personalized therapies^3^.

Metabolic reprogramming is regarded as a hallmark of malignant neoplasms, and a large number of investigations have demonstrated that the metabolic traits and predilections of tumors undergo alterations throughout the course of cancer development^4^. At the early stage, tumor growth requires a large amount of nutrient absorption and biosynthesis, and additional subtype-selective metabolic requirements arise during infiltration, which rely on new pathways for growth and metastasis^5^. Mitochondria are the key organelles involved in metabolic reprogramming in tumor cells, and maintaining mitochondrial integrity is the absolute basis of oxidative phosphorylation^6^. In addition, previous studies have also indicated that the number, structure, and function of mitochondria often change in malignant tumor cells to meet the needs of rapid growth in acidic and anoxic environments^7^.

As a key regulatory subunit of mitochondrial integrity, mitochondrial inner membrane protein (IMMT), also known as Mic-60 or Mitofilin^8^, is not only an important part of the mitochondrial contact sites and cristae organization system (MICOS) complex, but it also interacts with members of the SAM (The Sorting and Assembly Machinery) complex to form the mitochondrial membrane space bridging complex^9^. Recent studies have linked IMMT to the onset of various diseases, including cardiomyopathy^10^, degenerative encephalopathy^11^ and kidney injury^12^. However, research on the significance of IMMT in cancers is rare, and the only literature proposed that IMMT expression might be related to cancer prognosis^13,14^. To date, few reports have been published on the relationship between IMMT and the development and progression of BC, and the functions of IMMT in BC remains largely undefined.

The purpose of this investigation was to delve into the potential functions and underlying biological processes of IMMT in the development and progression of BC. To achieve this objective, bioinformatics and immunohistochemistry (IHC) were utilized to determine the prognostic significance of IMMT and its correlation with clinicopathological parameters in BC patients. Furthermore, in vitro experiments were conducted to assess the impact of IMMT on the proliferation and apoptosis of BC cells, and the morphological and functional changes of mitochondria after IMMT knockdown (KD) were also evaluated. Additionally, functional enrichment analysis and molecular-docking were performed to predict the possible mechanisms of IMMT involvement in BC progression.

## Materials and methods

### Bioinformatic analysis

Data on gene transcriptome and the corresponding clinical features of BC patients were conducted using The Cancer Genome Atlas (TCGA) (http://cancergenome.nih.gov/). In this cohort, 1096 samples from BC patients and 112 samples from healthy women were included. It was analyzed using the R package “limma” to compare gene expression levels between tumor and normal breast tissues. Based on IMMT expression in BC patients, survival analysis was performed using R programmes ‘survminer’ and ‘survival’. The association between clinicopathological factors and BC prognosis was assessed using a univariate and multivariate Cox analysis. A Gene Set Enrichment Analysis (GSEA) using the “Clusterprofile” package was performed on the TCGA database in order to investigate IMMT functions. The BC patients were equally divided into two groups according to the expression level of IMMT, and used FDR q < 0.1 and P < 0.05 to define significantly enriched functional annotations in the Kyoto Encyclopedia of Genes and Genomes (KEGG) pathways. Genes related to metabolic pathways were downloaded from the KEGG metabolic pathway on the GSEA website (brodinstitute.org), and predictions of IMMT binding to metabolic-related proteins were made through the STRING online tool (https://string-db.org).

### Kaplan–Meier survival analysis and human protein atlas (HPA)

The Kaplan–Meier tool (http://kmplotter.com) is an authoritative database for survival analysis. It uses meta-analysis combined with gene expression data and clinical prognosis information to assess the relationship between different genes and survival parameters. Using an online tool, we analyzed the breast cancer cohort for distant metastasis-free survival (DMFS), recurrence-free survival (RFS), and overall survival (OS). (OS: auto select best cutoff, excluding systemically untreated patients, with a follow-up period of 60 months). (DMFS: auto select best cutoff, excluding systemically untreated patients, with a follow-up period of 120 months). (RFS: auto select best cutoff, including all patients without restrictions on follow-up duration). The log P-value of the risk ratio and the 95% confidence interval were also calculated.

The HPA database (The Human Protein Atlas) is a comprehensive resource that provides information about the expression and function of human proteins (http://www.proteinatlas.org). Users can query the expression of specific proteins across different tissues, cells, and organs, as well as their differential expression in cancer. IMMT expression in normal breast tissues was compared with that in BC tissues based on data from the HPA.

### GEPIA and TIMER 2.0

GEPIA (https://GEPIA.cancer-PKU.cn/) is an online analytics website that provides fast and customizable analytics based on the TCGA and GTEx databases. The relationship between target gene expression and OS in patients with BC was estimated using GEPIA.

TIMER2.0 (http://timer.cistrome.org/) is an online analysis site that uses six state-of-the-art algorithms and provides four modules for studying associations between immune infiltrates and genetic or clinical features of cancer^15^. Comprehensive analysis and visualization functions have been provided for tumor research. Meanwhile, a TIMER2.0 analysis was performed to determine how IMMT is expressed in cancer tissues and normal tissues.

### Patients and tissue samples

A total of 130 paraffin-embedded tissues were collected from BC patients at the Affiliated Hospital of Zunyi Medical University. All patients were female diagnosed with invasive ductal carcinoma, and age 27–80 years (mean, 52.0 years). Clinicopathological information (e.g., tumor size, ER/HER-2 status, and Ki-67 level) was obtained from the medical records (Table 1). Prior patient consent and approval have been obtained from the Ethics Committee of Zunyi Medical University (approval No.2020-1-150), and the procedures followed were in accordance with the Helsinki Declaration.

**Table 1:** Clinicopathological features of patients with BC.

Characteristics	Number of cases (%)
Age (y)	
≤ 55	87 (66.9)
> 55	43 (33.1)
Menstrual state	
Pre-menopause	56 (43.1)
Post-menopausal	74 (56.9)
Histologic grade	
I	13 (10.0)
II	99 (76.2)
III	18 (13.8)
Tumor size	
T (≤ 2 cm)	28 (21.5)
T (> 2 cm)	102 (78.5)
Lymphatic metastasis	
No	55 (42.3)
Yes	75 (57.7)
Distant metastasis	
No	121 (93.1)
Yes	9 (6.9)
ER status^a^	
Negative	57 (43.8)
Positive	73 (56.2)
HER2 status^b^	
Negative	85 (65.4)
Positive	45 (34.6)
Ki-67 (%)^c^	
≤ 15%	29 (22.3)
> 15%	101 (77.7)
Molecular typing^d^	
Luminal A	15 (11.5)
Luminal B	57 (43.8)
HER-2	33 (25.4)
TNBC	25 (19.2)
IMMT expression	
Low expression	27 (20.8)
High expression	103 (79.2)

^a^ER positive interpretation standard: IHC nuclear staining ≥ 1%

^b^HER-2 positive interpretation criteria: IHC (3+) or IHC (2+) but FISH (fluorescence in situ hybridization) result was positive.

^c^Cutoff for Ki-67 is primarily referenced with 2011 St. Gallen consensus.

^d^The molecular typing was defined based on the 2011 St. Gallen consensus.

### IHC staining and scoring

An overnight fixation in formalin was followed by embedding the tumor specimen in paraffin and slicing it into 4 mm thick sections for IHC staining. With ethanol gradients, the slides were rehydrated after having been dewaxed in xylene. Following antigen retrieval, the slides were incubated with 3% hydrogen peroxide solution for 20 min. The slides were then washed 3 times for 5 min each time with phosphate-buffered saline (PBS) and blocked with 10% goat serum for 30 min at 37 °C. The Anti-IMMT antibody (ab137057; dilution, 1:500; Abcam) were incubated overnight at 4 °C, followed by a 1 h incubation with the secondary antibody (Servicabio, China). Following this, the tissue sections were visualized with DAB solution, and PBS was substituted for the primary antibody for a consistent negative control. Images were captured using an Olympus microscope (BX43). Based on the stained sections, two pathologists independently assessed and scored the sections as follows: cells with staining less than 10% were scored as +, 1; cells with 10–49% staining, ++, 2; cells with 50–74% staining, +++, 3; and cells with 75–100% staining, ++++, 4. Intensity of staining was recorded as no staining (0), light brown (1+), brown (2+), dark brown (3+). To calculate the IHC score for IMMT staining, multiply the tumor cell staining score by the staining intensity score. As defined above, low expression was 0–6 points, while high expression was above 6 points^16^.

### Cell culture

The normal breast cell line MCF-10A and breast cancer cell lines (MDA-MB-231, MDA-MB-436, MDA-MB-468, SK-BR-3, BT-549, MCF-7, T-47D, BT-474) were purchased from Procell Life Science & Technology Co., Ltd. (Wuhan, China) and cell bank of Chinese Academy of Sciences (Shanghai, China). All cells were incubated in standard incubator conditions (95% air, 5% carbon dioxide, and 37 °C) and recommended medium, according to the supplier’s specifications. Short tandem repeat profiling was used to confirm the authenticity of the above cells, and no mycoplasma contamination was observed (Mycoplasma test kit, CA1080, Solarbio).

### siRNA transfection

siRNAs targeting IMMT mRNA and a control siRNA were designed and synthesized by GenePharma (Shanghai, China). SK-BR-3 and MDA-MB-436 cells were seeded in 6-well plates and transfected for 72 h with corresponding siRNA using GP-transfect-MATE transfection reagent (Gene Pharma, China). Whole protein was extracted for verification 96 h after transfection. The sequences of the siRNAs used for IMMT-KD and control were as follows: 5′-CCGGGAAAGUGUAGAGAAATT-3′ and 5′-UUUCUCUACACUUUCCCGGTT-3′; 5′-UUCUCCGAACGUGUCACGUTT-3′ and 5′-ACGUGACACGUUCGGAGAATT-3′, respectively.

### Cell proliferation and clone formation

Proliferation ability of the cells was conducted according to the instructions using the Cell Counting Kit 8 assay (CCK-8; Solarbio, China). Transfect SK-BR-3 and MDA-MB-436 cells with si-Ctrl and si-IMMT. At 48 h post-transfection, seed the cells into a 96-well plate at a density of 8 × 10^3^ cells per well. Then, incubate the cells in the incubator and measure them at time points of 0, 24, 48, and 72 h. At each predetermined time point, gently remove the old medium from the 96-well plate and replace it with 100μL/well of fresh complete medium. Subsequently, add 10μL of CCK-8 solution to each well. Gently shake the plate to ensure thorough mixing of the CCK-8 solution with the medium, then place the plate back into the incubator for an additional 2-h incubation. After 2 h, use a microplate reader (ThermoFisher, USA) to measure the optical density (OD value) at 450 nm wavelength. Record the results to assess cell proliferation. Each group had five replicate wells. The data is presented as mean ± standard deviation (n = 3–4).

After transfecting SK-BR-3 and MDA-MB-436 cells with si-Ctrl and si-IMMT for 48 h, seed the cells into a 6-well plate with 3 replicate wells per group, at a density of 8 × 10^4^ cells per well. Culture the cells in complete medium containing 15% FBS for 2 weeks. After 2 weeks, remove the plate, discard the medium, and add 2 mL of methanol to each well to fix the cells at room temperature for 30 min. Then, discard the methanol and gently wash the wells with PBS. Subsequently, add 2 mL of 0.1% crystal violet solution to each well and stain for 3 min. Once staining is complete, wash with PBS to remove any excess dye. After rinsing, proceed to take photographs for documentation.

### Immunofluorescence, Mito-Tracker and TUNEL

SK-BR-3 cells were cultured for 48 h on a 24-well chamber slide, fixed with 4% paraformaldehyde for 30 min, followed by permeabilization with 0.1% Triton X-100 for 20 min at room temperature. Cells were blocked with 10% normal goat serum (cat. no. WGAR1009-5, Servicebio, Wuhan, China) for 1 h at room temperature and subsequently incubated with overnight at 4 °C with anti-IMMT (ab137057; dilution, 1:200; Abcam). The secondary, fluorescent antibody (711-095-152; dilution, 1:200; Jackson) would react with the primary antibody for 45 min in the dark at room temperature. For mitochondrial staining, SK-BR-3 was incubated with preheated (37 °C) medium containing Mito-Tracker Green (cat. no. C1048, Beyotime) for 45 min. Then it was fixed with 4% paraformaldehyde for 15 min, infiltrated with 0.1% Triton X-100, and blocked with 10% goat serum. Cells were incubated with cytochrome c primary antibody (136F3; dilution, 1:1000; CST), then with fluorescent secondary antibody, and finally with DAPI for nuclear staining. These images were taken under a fluorescence microscope (Olympus, Japan) and laser confocal microscope (ZEISS, Germany). The quantitative analysis of cytochrome c was based on immunofluorescence density of laser confocal electron microscopy images.

Apoptosis was detected by TUNEL apoptosis detection kit (cat. no. C1089, Beyotime) according to the instructions. Cells were stained for 15 min with DAPI at room temperature, and the images were captured under a fluorescence microscope (Olympus, Japan).

### Electron microscopy

The samples were immersed in a 2.5% glutaraldehyde solution (pH 7.4) for a duration of 2 h and subsequently embedded in agarose with a low melting point. Following this, the samples were subjected to three washes using a 0.1 M phosphate buffer (pH 7.2) and then fixed in a 1% osmic acid solution at a temperature of 4 °C for a duration of 2 h. The samples were then subjected to a gradient dehydration process using a series of ethanol solutions with varying concentrations. Subsequently, the samples were embedded in Epon-Araldite resin to facilitate penetration and placed in a model for polymerization. Ultrathin sections were collected from the samples for microstructure analysis. The sections were then counterstained using a 3% uranyl acetate and 2.7% lead citrate solution. Finally, the samples were observed using a HT7800 transmission electron microscope, and the number and average area of mitochondria were analyzed from 5 random fields of view, which were equal to the high magnification images of Transmission Electron Microscopy (TEM) in each group of samples.

### Western blot

Whole-cell lysis assay buffer was used to lyse the cells (cat. no. KGP2100. KeyGen Biotech, Jiangsu, China), and BCA protein assay kits (EpiZyme, Shanghai, China) were used to measure the protein concentrations. For each group, equal amounts of protein were separated using 10% SDS-PAGE and transferred to a polyvinylidene fluoride membrane (Merck Millipore, Germany). The membranes were incubated at room temperature for 2 h with 8% skim milk to block non-specific binding sites. The blocked membranes were incubated overnight at 4 °C with the following primary antibodies: IMMT (ab137057; dilution, 1:5000), Caspase3 (ab32351; dilution, 1:5000) and Caspase9 (ab32539; dilution, 1:5000) were purchased from Abcam. PCNA (PC10; dilution,1:800) and CCND1 (A-12; dilution, 1:800) were purchased from Santa Cruz Biotechnology. β-Actin (M1210-2; dilution, 1:5000) were purchased from HUABIO. Cleaved-Caspase-3 (5A1E; dilution, 1:1000), Cyt-c (136F3; dilution, 1:1000), Cleaved-Caspase-9 (D8I9E; dilution, 1:1000), Mitofusin-1 (D6E2S; dilution, 1:1000), Mitofusin-2 (D1E9; dilution, 1:1000), OPA1 (D7C1A; dilution, 1:1000), MFF (E5W4M; dilution, 1:1000), and Drp1 (D6C7; dilution, 1:1000), LDHA (C4B5; dilution, 1:1000), PFKP (D2E5, dilution, 1:1000), Hexokinase 1 (C35C4, dilution, 1:1000) were purchased from CST. Ki-67 (27309-1-AP, dilution, 1:2000) were purchased from Proteintech. Enhanced Chemiluminescence Detection Kit (MA0186; Meilunbio, Dalian, China) was used to visualize the signals after washing the membranes. Analyses of densitometry were conducted using Image-Pro Plus 6.0 software and normalized to β-Actin controls, and the final statistical analysis is based on the quantitative results of three independent grayscale values.

### Molecular docking

ZDOCK 3.0.2 was used to predict whether and how IMMT binds to individual metabolic-related proteins, as confirmed by the STRING tools. Before docking, the structural files of these proteins were obtained from the UniProt database. They were then treated with PyMol2.5.2 to remove water molecules, hydrogen atoms, and nontarget structural proteins. During docking, the default configuration of ZDOCK 3.0.2 was used for docking research, and global rigid docking was conducted. After docking, energy minimization was performed using AMBER18 under an ff14SB force field. Finally, the energy-minimized protein complex conformation was evaluated for binding energy using an online tool prodigy (https://wenmr.science.uu.nl/prodigy/), and the combinations based on the binding energy were visualized using PyMOL 2.5, including hydrogen bonding and salt bridging.

### Statistical analysis

All bioinformatics analyses of data downloaded from TCGA database were performed using R v.4.0.3. The TIMER, HPA, Kaplan–Meier, and GEPIA databases were used to generate expression and survival plots for the respective analyses, including hazard ratios (HR) and *P* values from the log-rank test. All data are presented as the mean ± standard error of the mean (mean ± SEM). Multiple groups were compared using a one-way analysis of variance followed by Tukey’s post-hoc test three times for each in vitro experiment. A minimum of three independent replicates were conducted for all cell experiments, yielding consistent results. Statistical analyses were performed using the SPSS software package (IBM, Version 29.0): The relationship between IMMT expression and clinicopathological features was analyzed using the chi-square test; molecular subtyping and histologic grade was assessed using the Kruskal Wallis test; ER status and HER-2 status were analyzed using the nonparametric Mann–Whitney test; all other results were analyzed using two-tailed unpaired Student's t-tests. Statistical significance was set at an *α*-value less than 0.05.

### Ethics statements

This study was approved by the Ethics Committee of Zunyi Medical University (approval No. 2020-1-150), and written informed consent was obtained from all patients.

## Results

### Upregulation of IMMT in BC is associated with poor prognosis

**Figure 1:**
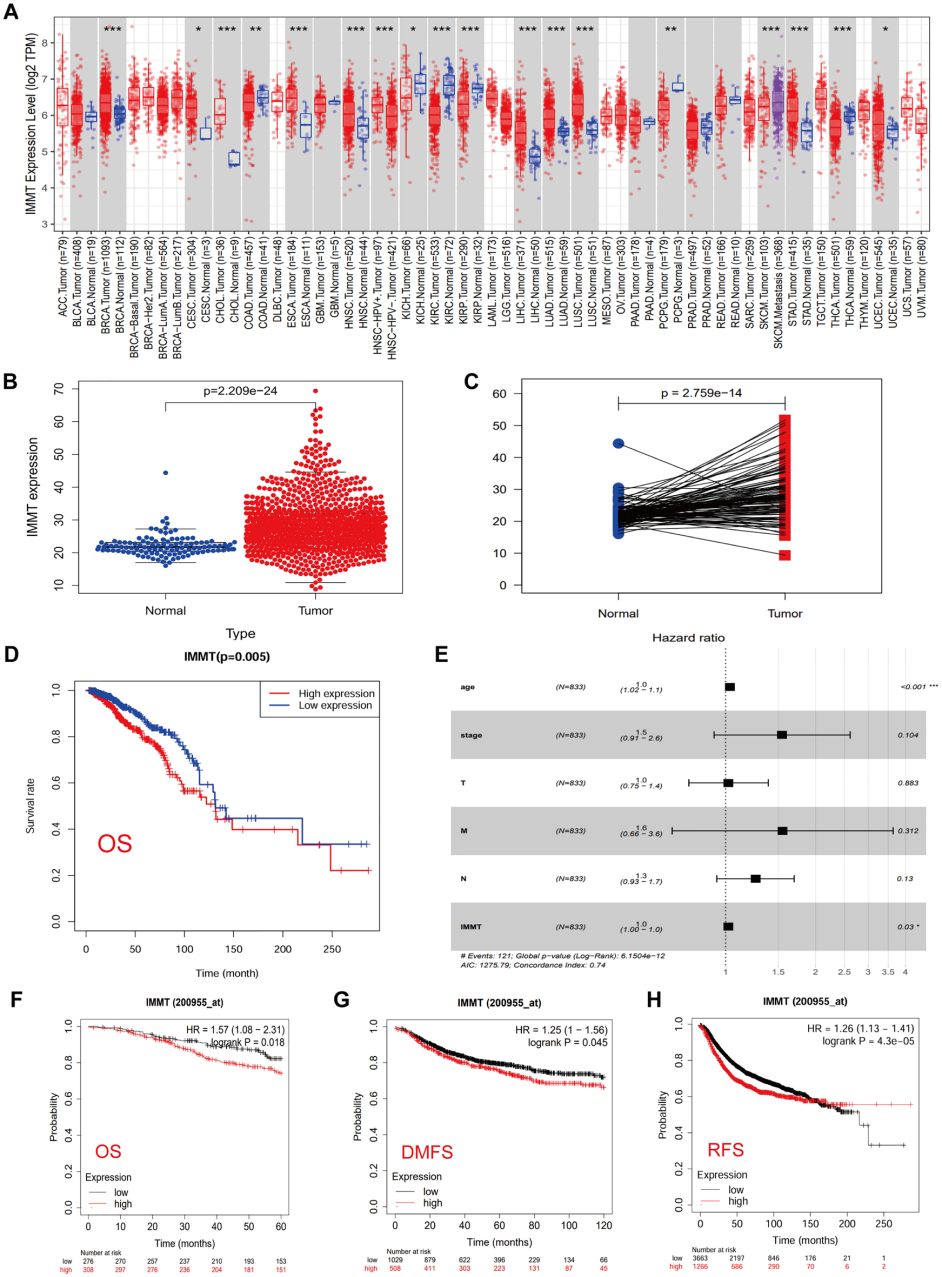
IMMT is highly expressed in BC and is related to poor prognosis. (**A**) An analysis of IMMT expression in TIMER2.0 database between tumor tissues and normal tissues (*P < 0.05, **P < 0.01 and ***P < 0.001). (**B**) Based on the TCGA database, IMMT is expressed in BC and normal breast tissues. (**C**) The expression of IMMT in paired BC and normal breast tissues based on TCGA database. (**D**) Kaplan–Meier survival analysis of BC patients based on IMMT mRNA expression levels. (**E**) Prognostic significance of IMMT in BC patients assessed by multivariate Cox analysis. (**F**–**H**) This is supported by Kaplan–Meier analysis, which indicates that heightened IMMT levels are linked to poorer outcomes in terms of overall survival (OS), distant metastasis-free survival (DMFS) and relapse-free survival (RFS).

The mRNA expression of IMMT was assessed across multiple cancer types using the TIMER 2.0 dataset. Figure 1A illustrates that the mRNA expression of IMMT varies among different cancer types, with a notably elevated level observed in BC tissue compared to normal tissue. Similarly, differential expression analysis through the TCGA dataset also revealed that a higher level of IMMT gene expression was found in BC tissues compared with normal breast tissues (Fig. 1B). To eliminate the interference of unequal numbers of normal and cancerous tissues in the data samples, paired analysis was performed, and the results also showed that BC samples expressed significantly higher levels of IMMT (Fig. 1C). Subsequent Kaplan–Meier analysis demonstrated that the survival time in the IMMT high expression group was significantly shorter than that in the low expression group (Fig. 1D). According to uni-and multivariate Cox regression analysis of IMMT gene expression and clinicopathological factors (Table S1), the expression of IMMT in BC may be an independent prognostic factor for overall survival. (Fig. 1E). Further investigation of the prognostic value of IMMT in BC using the online survival analysis tool (http://kmplot.com/analysis/) showed similar results to the TCGA database, high levels of IMMT expression were associated with worse OS (HR = 1.57,* P* = 0.018), DMFS (HR = 1.25, *P* = 0.045), and RFS (HR = 1.26, *P* = 4.3e−0.5) in BC patients (Fig. 1F–H), respectively.

### High expression of IMMT is associated with high-risk clinicopathological factors in BC

**Figure 2:**
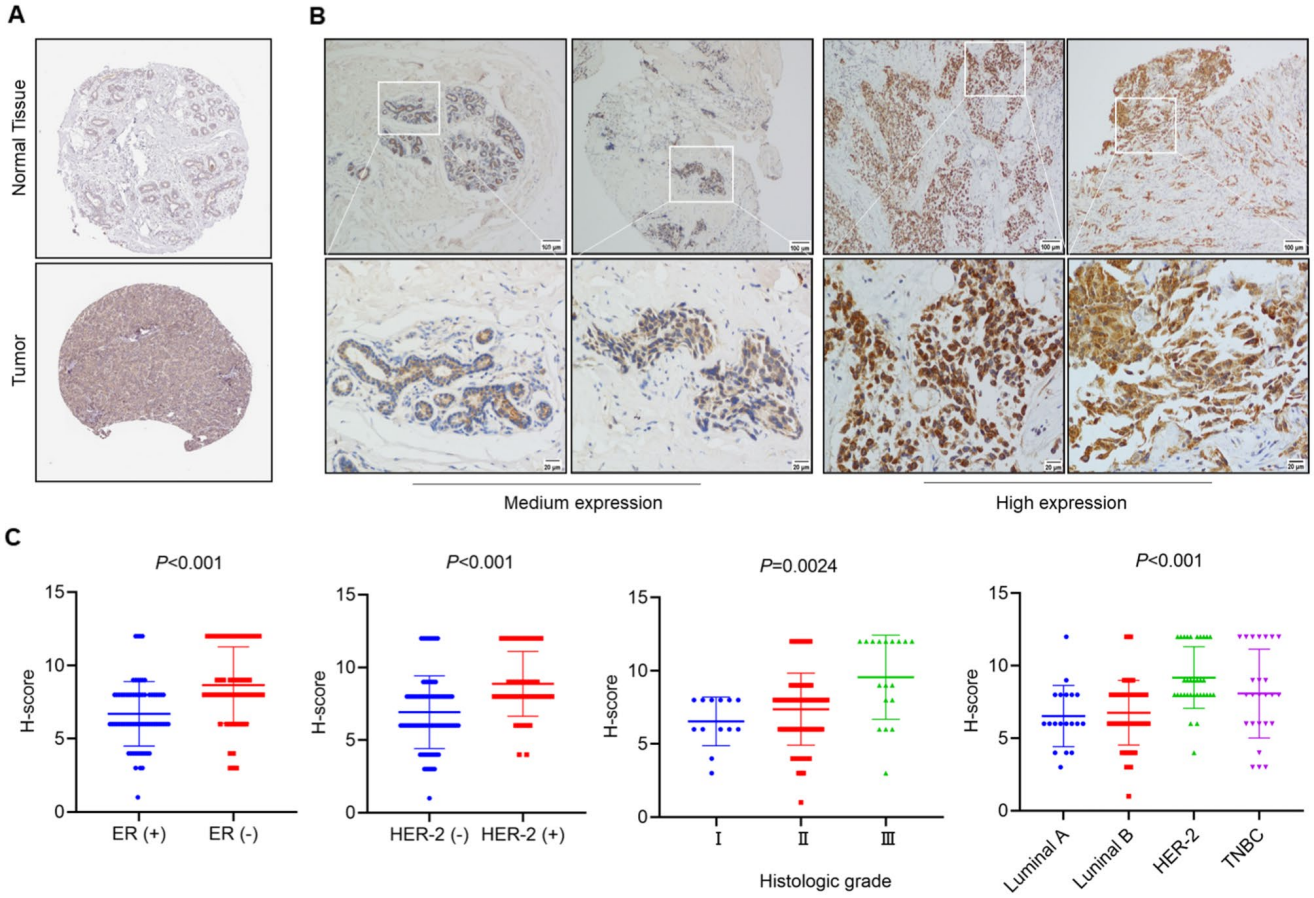
The high expression of IMMT in BC tissues is significantly related to adverse prognosis. (**A**) Analysis of IMMT protein expression levels in normal breast tissue and tumor tissue through the HPA database. (**B**) Expression level of IMMT in BC tissues. (**C**) According to the Mann–Whitney test, IMMT exhibits higher expression levels in tumor tissues that are ER-negative and HER-2 positive; assessment by the Kruskal Wallis test indicates significant differences in IMMT expression across various histological grades and molecular subtypes.

Following analysis using the HAP database to assess IMMT protein expression differences between normal and cancerous breast tissues, it was found that IMMT exhibited high expression in BC tissues, contrasting with moderate to low expression levels in normal breast tissues (Fig. 2A). IHC staining demonstrated prominent IMMT expression in the labeled cytoplasmic granules of BC cells (Fig. 2B). A high prevalence of increased IMMT expression was observed in 103 out of 130 tumors (79.2%), as detailed in Table 2. Using analysis of variance (ANOVA), a significant correlation was found between high IMMT expression and larger tumor size (> 2 cm, *P* = 0.028), increased Ki-67 index (> 15%, *P* = 0.039), and positive HER-2 status (*P* = 0.004) (Table 2). Additionally, we conducted further analysis using the nonparametric Mann–Whitney test to assess ER and HER-2 status, and the Kruskal Wallis test was employed for molecular subtyping and histologic grade. Our analysis revealed that IMMT expression is relatively higher in tumor tissues that are ER-negative and HER-2 positive (*P* < 0.001), and this elevated IMMT expression correlates with a higher histologic grade of the tumor (*P* = 0.0024) and is closely associated with molecular subtypes of BC (*P* < 0.001) (Fig. 2C). These findings suggest an association between high IMMT expression and adverse clinicopathological traits in patients diagnosed with BC.D

**Table 2:** Association between IMMT expression and clinicopathological features in BC patients.

Characteristics IMMT	IMMT		*P* value
	Low expression	High expression	
Age (y)			0.669
≤ 55	19	68	
> 55	8	35	
Menstrual state			0.301
Pre-menopause	14	42	
Post-menopausal	13	61	
Histologic grade			0.552
I	3	10	
II	22	77	
III	2	16	
Tumor size			0.028
T (≤ 2 cm)	10	18	
T (> 2 cm)	17	85	
Lymphatic metastasis			0.49
No	13	42	
Yes	14	61	
Distant metastasis			0.753
No	26	95	
Yes	1	8	
ER status			0.094
Negative	8	49	
Positive	19	54	
HER2 status			0.004
Negative	24	61	
Positive	3	42	
Ki-67(%)			0.039
≤ 15%	10	19	
> 15%	17	84	
Molecular typing			0.003
Luminal A	8	7	
Luminal B	11	46	
HER-2	2	31	
TNBC	6	19	

### IMMT knockdown inhibits the proliferation of BC cells

**Figure 3:**
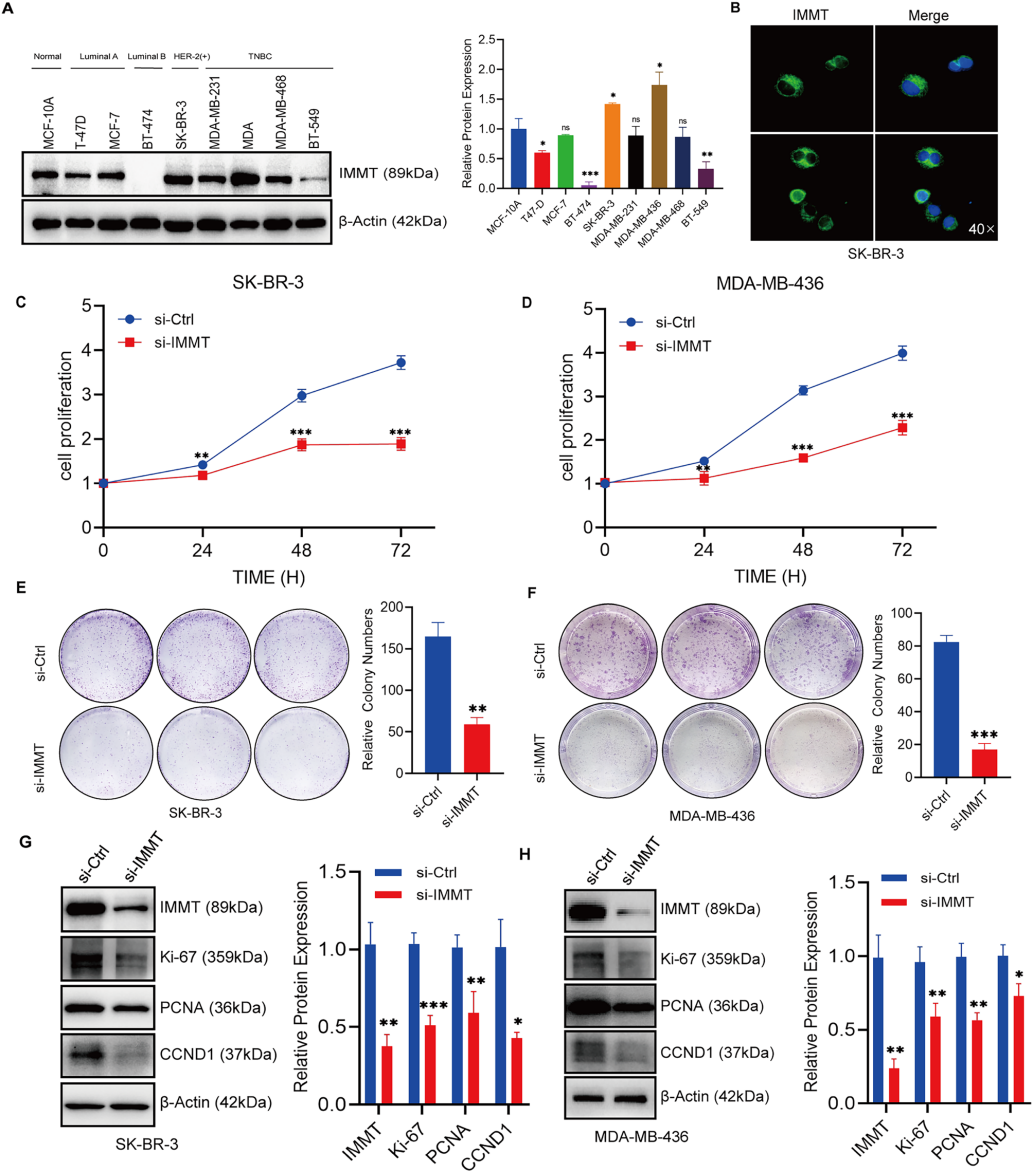
The proliferation of BC cells is inhibited in the absence of IMMT. (**A**) This is supported by the expression of IMMT protein in various types of BC cell lines. Mean ± SD (n = 3). (**B**) The immunofluorescence localization of IMMT in SK-BR-3 cells. (**C**,**D**) CCK-8 assays revealed suppressed proliferation in SK-BR-3 and MDA-MB-436 cells upon knockdown of IMMT. Mean ± SD (n = 3 to 4). (**E**,**F**) The decrease in colony counts of SK-BR-3 and MDA-MB-436 cells transfected with si-IMMT compared to the si-Ctrl group. (**G**,**H**) This was confirmed through Western blot analysis of proliferation-related proteins following si-IMMT transfection. Data are presented as mean ± SEM, *P < 0.05, **P < 0.01 and ***P < 0.001.

In order to further explore the biological function of IMMT, we detected its expression in various types of BC cells. As shown in Fig. 3A, IMMT protein expression was the highest in MDA-MB-436 and SK-BR-3 cells, whereas lowest expression was observed in BT-474 cells. Next, we selected SK-BR-3 and MDA-MB-436 cells for further experimental verification. According to Fig. 3B, the immunofluorescence results revealed that IMMT was expressed in the cytoplasm, consistent with its localization in the mitochondria. The IHC analysis of clinical tissues confirmed that the IMMT expression was correlated with a high Ki-67 index, and thus, we speculated that IMMT could act as a regulator of BC cell proliferation. To examine the aforementioned hypothesis, we employed si-RNA to suppress the expression of IMMT. The outcomes derived from CCK-8 and clone formation experiments demonstrated a notable decrease in the proliferation and clone formation capacity of BC cells subsequent to the IMMT-KD (Fig. 3C–F). Additionally, alterations in the expression levels of proliferation-associated proteins were assessed. As depicted in Fig. 3G,H, a substantial reduction in the expression of Ki-67, PCNA and CCND1 being proliferation-related proteins, was observed following the IMMT-KD.

### The impact of IMMT on mitochondrial dynamics and the mitochondrial apoptosis pathway

**Figure 4:**
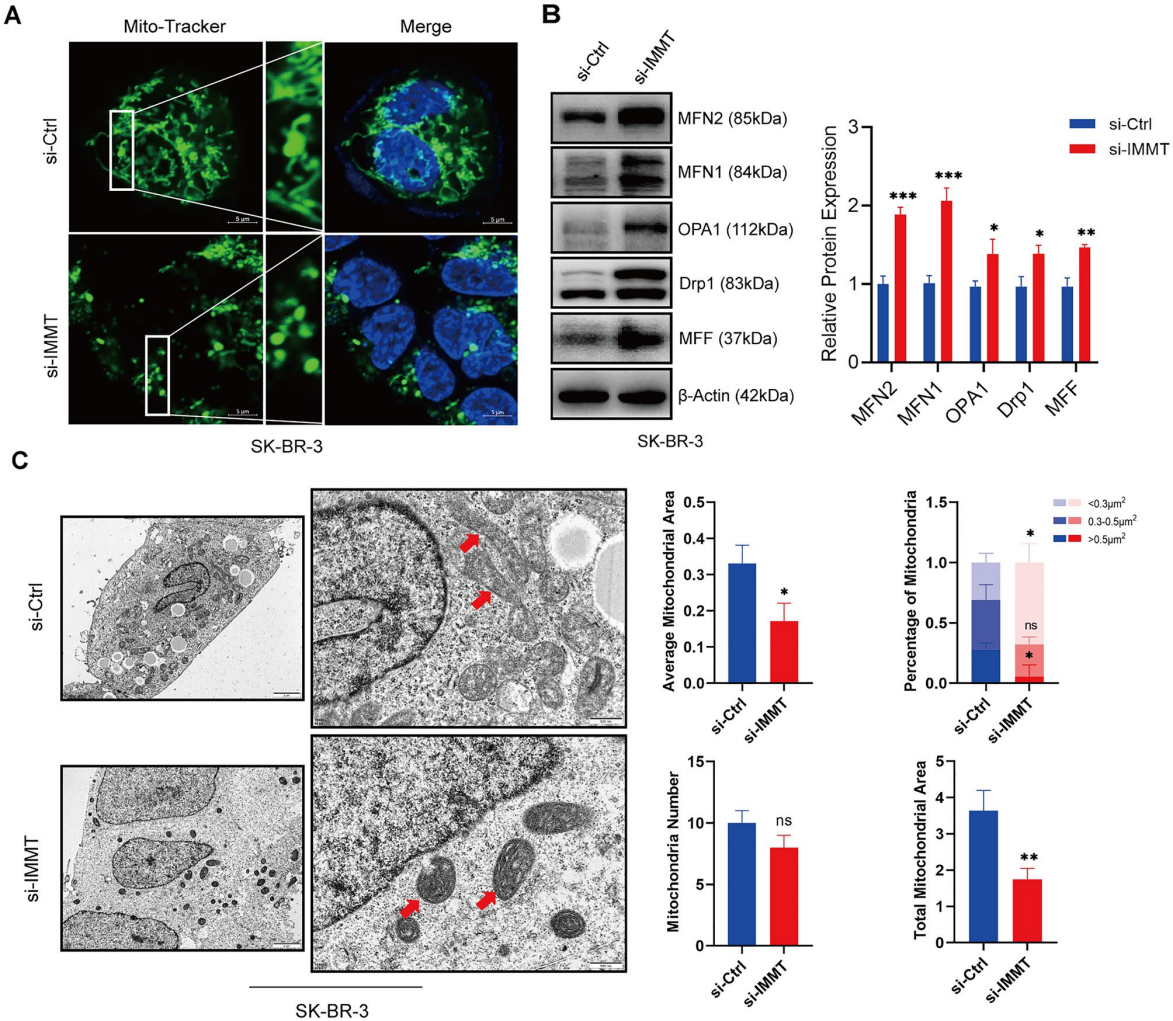
The depletion of IMMT results in alterations to the morphology of mitochondria. (**A**) SK-BR-3 cells were observed with laser confocal after IMMT-KD to observe Mito-Tracker staining. (**B**) The alteration of mitochondrial dynamic proteins subsequent to the deletion of IMMT was evaluated through immunoblotting analysis. (**C**) SK-BR-3 cells transfected with si-IMMT were analyzed by transmission electron microscope, and the number and area of mitochondria were analyzed and counted (the red arrows indicate mitochondria). Data are presented as mean ± SEM, *P < 0.05, **P < 0.01 and ***P < 0.001.

Related studies have proved that IMMT, as the core subunit of MICOS, is very important to the structure of mitochondrial cristae^17^. To further investigate the impact of IMMT deletion on mitochondria, Mito-Tracker staining was conducted to visualize the alterations in mitochondria, revealing a distinct transformation from elongated tubular structures to short dotted tubular structures (Fig. 4A). Then the examination of proteins related to mitochondrial dynamics revealed that the downregulation of IMMT leads to an increase in the levels of proteins involved in mitochondrial fusion (MFN2, MFN1, OPA1) and fission (DRP1, MFF) (Fig. 4B). Additionally, examination through transmission electron microscopy unveiled onion-like and lamellar change in the mitochondria of IMMT-KD cells, compared with the control group which exhibited the characteristic elongated tubular morphology, further quantitative statistical analysis shows that although there is no star difference in the number of mitochondria, compared with the control group, the number of small mitochondria (< 0.3 mm^2^) in the IMMT low expression group is obviously increased (Fig. 4C).

**Figure 5:**
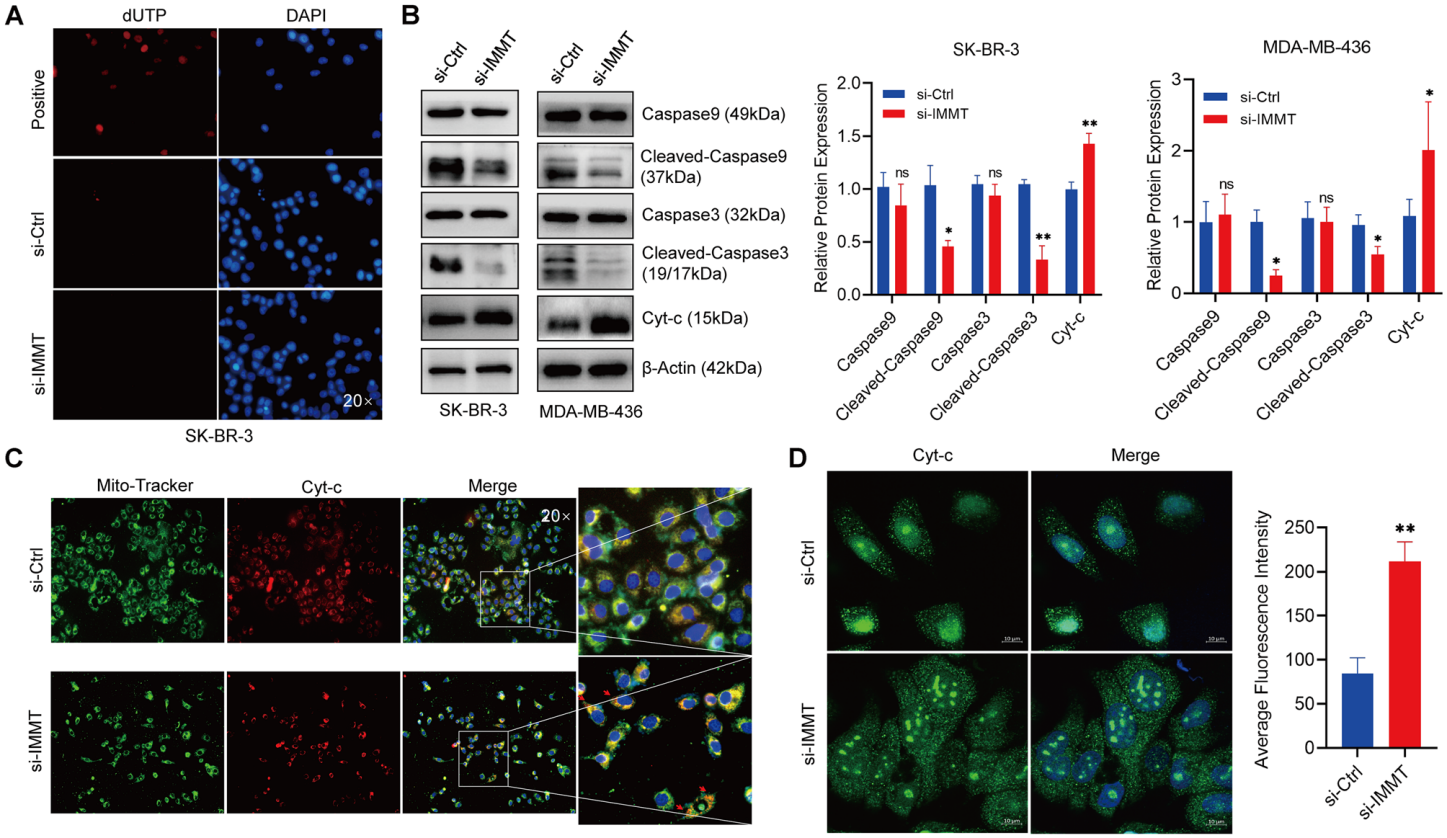
IMMT deficiency leads to the release of cytochrome c, but does not induce mitochondrial apoptosis. (**A**) TUNEL was used to detect apoptosis after treating cells with si-IMMT for 48 h, the positive control was treated with 10 nM paclitaxel for 24 h. (**B**) The expression of apoptosis-related proteins was detected by Western-blot. (**C**,**D**) Following the knockdown of the IMMT, the localization of cytochrome c was examined using a fluorescence microscope (the red arrow indicates cytochrome c) and laser confocal microscope. Data are presented as mean ± SEM, *P < 0.05, **P < 0.01.

In the preceding step, it was demonstrated that the inhibition of IMMT effectively impedes cell proliferation and induces alterations in mitochondrial morphology. Consequently, an investigation into the mitochondrial apoptosis pathway was conducted to ascertain whether the IMMT-KD triggers its activation. The results showed that although the knockdown of IMMT partially affected the cell proliferation, no apoptosis signal was detected in our experimental results (Fig. 5A), and the detection of apoptosis-related proteins also showed that the mitochondrial apoptosis pathway was not obviously activated (Fig. 5B). In addition, we found that cytochrome c and mitochondria are co-stained, and the lack of IMMT will lead to a large release of cytochrome c (Fig. 5C). The change of cytochrome c positioning under laser confocal microscope further proves this phenomenon (Fig. 5D). This release may be attributed to the destruction of mitochondrial crista morphology, because cytochrome c is mainly located in mitochondria. Interestingly, as a key factor of programmed cell death, this release did not obviously induce mitochondrial apoptosis pathway, and further research needs to clarify the precise potential mechanism.

### IMMT is involved in metabolic regulation in BC

**Figure 6:**
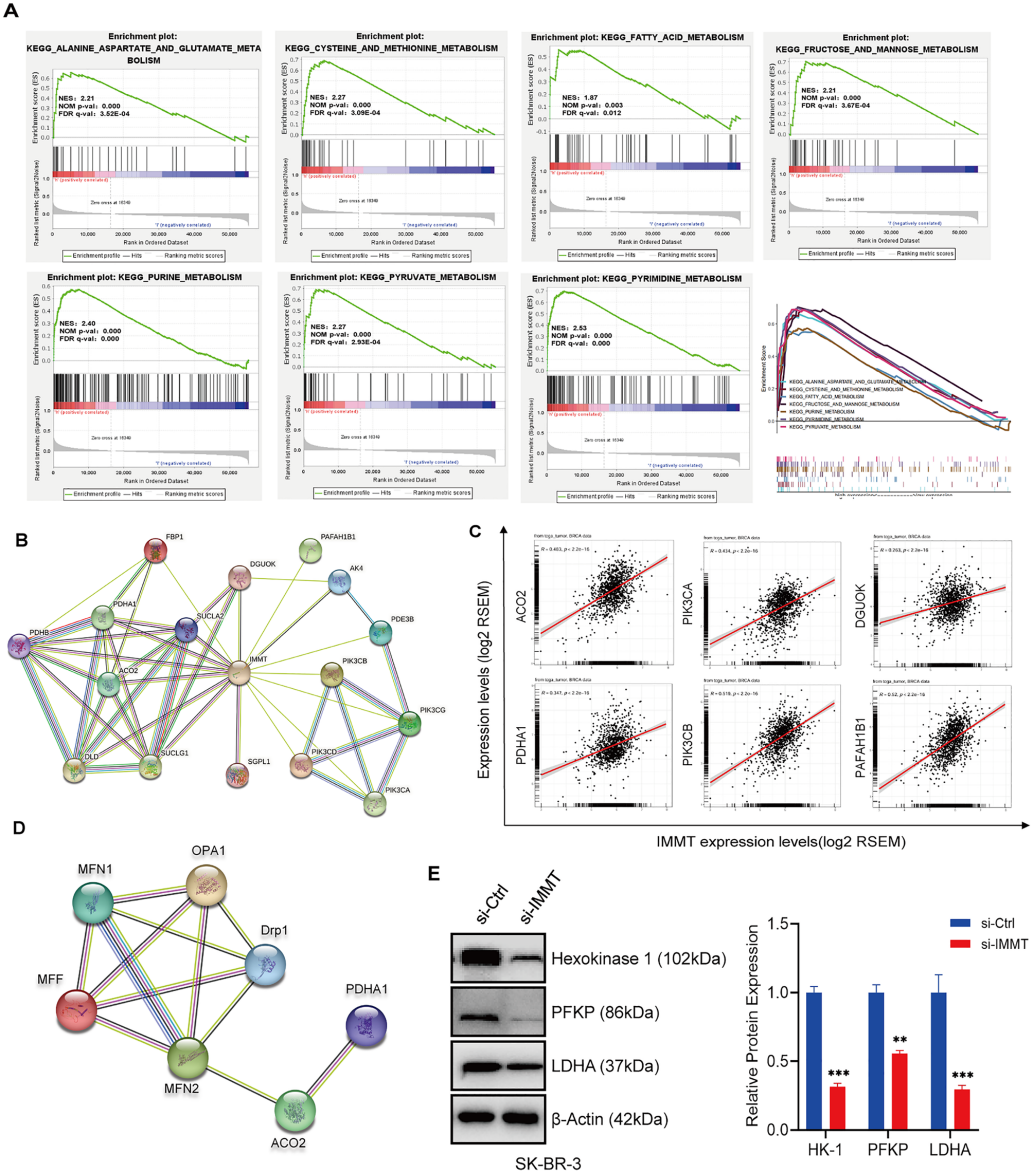
Gene set enrichment analysis (GSEA) based on the expression of IMMT in BC patients from TCGA database. (**A**) GSEA results show significant enrichment of metabolic pathways in BC patients with high expression of IMMT. Seven metabolic-related pathways enriched in IMMT high expression group NES, normalized enrichment score. (**B**) The interaction network between IMMT and metabolic-related proteins was generated using STRING. (**C**) Correlation analysis between IMMT and six interacting molecules: ACO2, PAFAH1B1, PDHA1, DGUOK, PIK3CA, and PIK3CB. (**D**) Interaction networks of ACO2 and PDHA1 with mitochondrial dynamics-related proteins (MFN2, MFN1, MFF, Drp1, OPA1). (**E**) Western blot detected glycolysis related indexes after IMMT-KD. Data are presented as mean ± standard error of the mean, *P < 0.05, **P < 0.01 and ***P < 0.001.

To gain a deeper understanding of the role of IMMT in BC, we conducted an analysis of the enrichment pathway of IMMT differential expression in BC using the TCGA database. Interestingly, the results revealed that a considerable number of metabolic pathways were enriched in the subgroup with high IMMT expression (Table S2). Specifically, these pathways completely contained the metabolism of almost all important substances, including alanine, aspartic acid, and glutamate, cysteine and methionine, fatty acid, fructose and mannose, purine, pyruvate, and pyrimidine (Fig. 6A). Other relevant enriched metabolic pathways, including methyl butyrate metabolism, galactose metabolism, tryptophan metabolism, porphyrin and chlorophyll metabolism, and amino sugar and nucleotide sugar metabolism, are listed in Table S2. The above analysis suggests that IMMT may be involved in the metabolic regulation of BC.

Furthermore, we made predictions and conducted an analysis on the molecules associated with metabolism that have the potential to interact with IMMT. A total of 16 molecules associated with metabolism were discovered to potentially engage in direct or indirect interactions with IMMT (Fig. 6B), and these genes were involved in the regulation of glycolysis (DLD, PDHB, and PDHA1), tricarboxylic acid (TCA) cycle (ACO2, SUCLA2, and SUCLG1), gluconeogenesis (FBP1), lipid metabolism (SGPL1 and PAFAH1B1), energy metabolism (DGUOK and AK4), and phosphatidylinositol 3- kinase protein family and their downstream factors (PIK3CA, PIK3CB, PIK3CD, PIK3CG, and PDE3B) (Table S3). Subsequent analysis of the correlation between these interacting molecules in BC using TCGA database revealed that 93.8% (15/16) of metabolic-related genes were correlated with IMMT (data not shown). Figure 6C specifically highlights the robust positive correlation between IMMT and these six genes (ACO2, PAFAH1B1, PDHA1, DGUOK, PIK3CA, and PIK3CB), indicating their potential significance in BC metabolism. In order to further clarify whether IMMT can regulate cell metabolism through mitochondrial dynamics, the interaction between dynamic proteins, ACO2 and PDHA1 was analyzed, and it was found that there was a close correlation between them (Fig. 6D). Finally, we detected changes in glycolysis related pathways in SK-BR-3 cell lines and found that the key rate limiting enzyme in glycolysis HK-1, PFKP and LDHA was significantly inhibited after IMMT-KD (Fig. 6E). These findings suggest that IMMT may regulate the metabolism of BC.

### Protein binding prediction based on molecular docking

**Figure 7:**
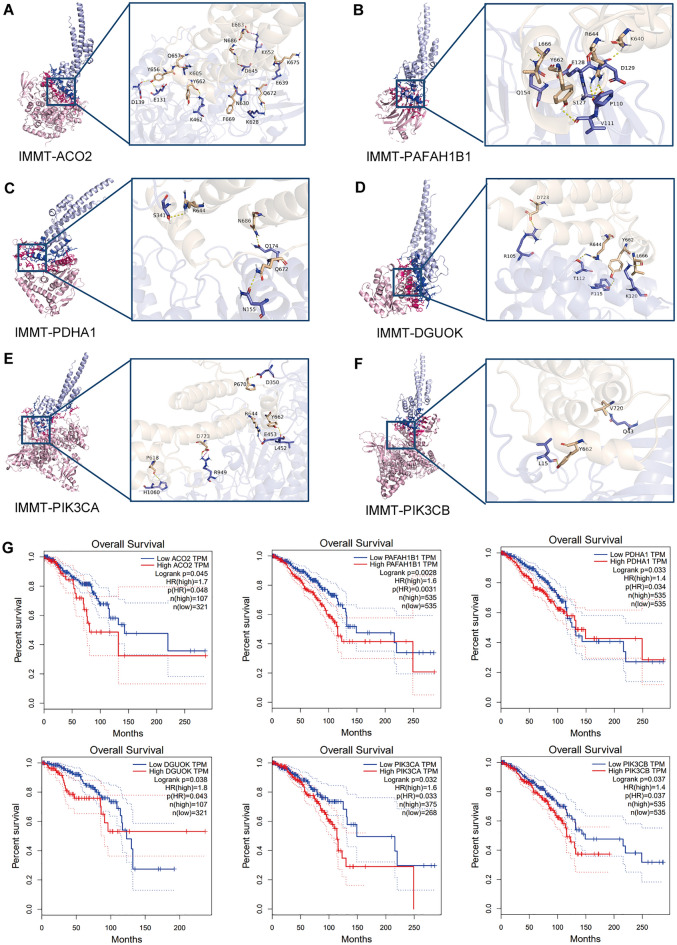
The present study investigates the 3D molecular docking interaction between IMMT and its related ligands. (**A**–**F**) The binding mode of the IMMT-ACO2, IMMT-PAFAH1B1, IMMT-PDHA1, IMMT-DGUOK, IMMT-PIK3CA, and IMMT-PIK3CB complexes is analyzed, with IMMT represented in wheaten and the target protein in blue. Hydrogen bonding and hydrophobic interactions are respectively denoted by yellow and wine red dotted lines. (**G**) The overall survival of 6 IMMT-associated metabolic genes in breast cancer patients were analyzed with GEPIA.

Protein interactions play an important role in signal transduction, gene expression, antibody-antigen complexes, and other biological processes^18,19^. Therefore, exploring protein interactions helps us to understand the specific effects of different factors. Based on the protein binding predictions above, we predicted the protein–protein binding affinities of IMMT with the 16 interacting proteins. The results showed that the combined values of binding affinity were all negative, indicating that binding was possible (Table 3). Concurrently, we further predicted the possibility of binding to IMMT by molecular docking of the six most relevant molecules in the above proteins. The results showed that all the target proteins were bound to the binding bags of IMMT (Fig. 7A–F). Further analysis of the binding patterns and binding forces between these proteins to predict their binding sites using ZDOCK 3.0.2 prediction showed that the docking of these proteins was mainly completed through hydrogen bonds and salt bridges (Table S4), which further verified the possibility of the above protein to bind on the molecular structure. Moreover, survival analysis of these genes also showed that high expression of ACO2, PAFAH1B1, PDHA1, DGUOK, PIK3CA, and PIK3CB in BC was significantly associated with poor OS (Fig. 7G). Based on these findings, IMMT may affect BC patients’ prognosis through interactions with these metabolism- related genes.

**Table 3:** Docking-derived binding affinity of IMMT with interacting metabolic related proteins.

System	Binding energy (kcal/mol)
IMMT-SGPL1	− 15.4
IMMT-PIK3CG	− 14.6
IMMT-ACO2	− 14.2
IMMT-PAFAH1B1	− 13.5
IMMT-AK4	− 12.6
IMMT-PDE3B	− 12.2
IMMT-PDHA1	− 11.6
IMMT-PIK3CD	− 11.5
IMMT-PIK3CA	− 11.4
IMMT-DGUOK	− 10.1
IMMT-SUCLA2	− 9.8
IMMT-PDHB	− 9.4
IMMT-DLD	− 9.3
IMMT-PIK3CB	− 9.3
IMMT-FBP1	− 9
IMMT-SUCLG1	− 8.3

## Discussion

This study demonstrated the differential expression of IMMT in human cancers. The expression of IMMT in BC tissues was higher than that in healthy breast tissues. Moreover, patients with BC exhibiting IMMT upregulation were associated with poor prognoses. Interestingly, our survival analysis results revealed a phenomenon of convergence and even crossing of survival curves after a 10-year follow-up. Of course, changes in patient management, disease progression, or other confounding variables that may arise later in the follow-up period are all factors that could influence this trend. Analysis of clinical information revealed that IMMT upregulation was correlated with tumor size (> 2 cm), Ki-67 expression (> 15%), and advanced histological grade. These results indicate that IMMT is a potential prognostic marker for BC. The findings of this study were consistent with those of Lin et al*.*^20^. However, this study reports a significant variation in the expression of IMMT in BC tissues based on different HER-2 statuses, which has not been documented before. This suggests that IMMT may play a role in the pathogenesis of HER-2 positive breast cancer, or there may be a certain correlation between IMMT and the HER-2 signaling pathway, and further research is needed to validate this hypothesis.

IMMT, component of the MICOS complex, has critical biological roles^21^. It not only participates in the formation and maintenance of contact points (CJs) between mitochondrial cristae but can also form an intermembrane space bridging complex with the SAM complex for signal transmission.^22^. Additionally, IMMT regulates the biological behavior of tumor cells by modulating the dynamic processes of mitochondrion division, fusion, transportation, degradation, and biogenesis^23,24^. Therefore, this study further investigated the function of IMMT using in vitro experiments. Transfection with siRNA targeting IMMT suppressed the proliferation of SK-BR-3 and MDA-MB-436 cells and significantly downregulated the expression levels of proliferation-related proteins, such as CCND1 and PCNA. Additionally, IMMT-KD significantly downregulated the expression of Ki-67, which validated the findings of clinical tissue analysis. The results of in vitro experiments were consistent with those of Ghosh et al.^25^ who reported that IMMT-KD decreases cell proliferation ability and promotes G2/M DNA content in pancreatic cancer cell lines. This inhibition of cell proliferation may be due to the downregulation of IMMT, which leads to a lot of mitochondrial damage, and then the cell program tries to avoid cell death, thus slowing down cell proliferation^25^.

The integrity of the mitochondrial structure is closely related to its biological functions, including the generation and transportation of energy and the transduction of cytoplasmic signals^26,27^. Therefore, the effect of IMMT-KD on the mitochondrial structure was examined. Through Mito-Tracker staining, it can be observed that after IMMT-KD, the mitochondria in cells undergo a transformation from an elongated filamentous structure to a punctate form, indicating enhanced mitochondrial fission activity. Consistently, transmission electron microscopy revealed the onion-like changes and cristae remodeling of mitochondria in IMMT-KD cells. Although the quantity of IMMT-KD mitochondria did not significantly decrease, compared to the control group, there was a nearly complete disappearance of large mitochondria and an observed increase in the number of small mitochondria. This finding is similar to that of Clark et al.^28^ who reported that IMMT mutation leads to changes in mitochondrial movement and ultrastructure in the drosophila model. Another interesting finding in our research is that mitochondrial dynamic proteins, such as MFN1, MFN2, MFF, OPA1, and Drp1, were upregulated upon IMMT-KD, which indicated the upregulation of mitochondrial fusion and division and the downregulation of mitochondrial mass. The mechanisms underlying these processes needs further research.

The destruction of mitochondrial cristae, which serve as the repository of cytochrome c, promotes its release, which could induce the activation of the mitochondrial apoptosis pathway. This study demonstrated that IMMT-KD promoted cytochrome c release from the mitochondrial cristae using fluorescence localization. However, the results of the terminal deoxynucleotidyl transferase biotin-dUTP nick end labeling assay did not reveal the induction of apoptosis or the activation of the caspase apoptosis system. These findings are consistent with those of Yang et al*.*^29^ who reported cytochrome c release in the absence of BAX activation. Interestingly, OPA1 is essential for maintaining mitochondrial cristae structure and preventing the release of cyt-c^30^. However, our findings surprisingly reveal that OPA1 upregulation coincides with cyt-c release. This seemingly contradictory observation could be explained by two possible mechanisms. First, it may be due to the accumulation of GTPase and the mitochondrial dynamics-related protein Drp1 within the mitochondria, leading to excessive mitochondrial fission, which disrupts their structure and function, thereby triggering the release of cyt-c^31,32^. Secondly, this contradiction might be the result of a severe stress response caused by the lack of IMMT. Although cells attempt to compensate for this stress and maintain mitochondrial homeostasis by increasing OPA1 expression levels, this effort appears to be limited, leading to the unusual concurrent occurrence of high OPA1 expression levels and cytochrome c (cyt-c) release. Of course, this conclusion requires further verification. Previous studies have reported that during apoptosis, mitochondria undergo fragmentation before caspase activation, resulting in the release of pro-apoptotic factors^33^. The upregulation of mitochondrial fission inhibits apoptosis by regulating the NF-κB and p53 signaling pathway^34^. These also might be the reason why we did not detect caspase activation in this study. However, mitochondrial dynamics vary in different cell types. The role of mitochondria in cancer development is complex and has not been elucidated. Further studies are needed to investigate the effect of IMMT on the biological behaviors of BC and the underlying mechanisms.

Mitochondria, which are the core of the cellular metabolic network, regulate cell metabolism and provide key metabolites for macromolecule synthesis, enabling the maintenance of tumor phenotype and promoting tumor growth^35,36^. To examine the potential mechanism of IMMT in BC, TCGA dataset was subjected to gene set enrichment analysis. KEGG analysis revealed that the differentially expressed genes were mainly enriched in pathways related to metabolic regulation. These pathways, including glycolysis, gluconeogenesis, tricarboxylic acid (TCA) cycle, lipid metabolism, and energy metabolism, account for the metabolism of almost all important substances.

Next, the mechanisms involved in IMMT mediated metabolic regulation in BC were examined. Protein–protein interaction network was constructed using the STRING database. In total, 16 metabolism-related proteins directly or indirectly interacted with IMMT. These molecules can be divided into the following five categories: phosphatidylinositol 3-kinase protein family and its downstream factors (PIK3CA, PIK3CB, PIK3CD, PIK3CG, and PDE3B), glucose metabolic regulators (DLD, PDHB, PDHA1, and FBP1), TCA cycle regulators (ACO2, SUCLA2, and SUCLG1), lipid metabolism regulators (SGPL1 and PAFAH1B1), and energy metabolism regulators (DGUOK and AK4), and the binding of IMMT to these molecules was confirmed using molecular docking. Cancer cells prefer to increase the rate of glycolysis rather than the TCA cycle to rapidly gain energy through glycolytic enzymes and glucose transporters, resulting in the upregulation of lactic acid levels under normoxic conditions (Warburg effect)^37^. Notably, through further STRING analysis, we found that mitochondrial dynamics-related proteins (MFN2, MFN1, MFF, Drp1, OPA1) are related to ACO2 and PDHA1. It was also found that the key rate-limiting enzyme in glycolysis HK-1, PFKP and LDHA were significantly inhibited after IMMT-KD. Therefore, we suggest that the down-regulation of IMMT expression may inhibit glycolysis by changing mitochondrial dynamics, thus inhibiting BC cell proliferation.

In conclusion, our research demonstrated that IMMT is a potential prognostic marker for BC and IMMT-KD effectively suppresses the proliferation of BC cells and offered valuable insights into the potential mechanism underlying the regulation of BC metabolism. However, this study has some limitations. First, the clinical validation in this study was mainly based on the data from a single center and multicenter studies must be performed to verify the findings of this study. To ensure that our results are robust and applicable across different patient populations and treatment settings, multicenter studies must be performed to verify the findings of this study. Future research should aim to expand our sample size, which could lead to a better understanding of the role that IMMT plays in cancer biology and its clinical significance. Additionally, the mechanisms of IMMT in metabolic regulation were mainly established using bioinformatics analysis. The specific role of IMMT in the metabolic regulation of BC needs further experimental verification.

## Conclusions

Through bioinformatics and clinical correlation analysis, we found that the high expression of IMMT in BC is closely related to high-risk clinicopathological factors and poor prognosis, suggesting that IMMT may be a prognostic marker of BC. IMMT-KD inhibited the proliferation of BC cells accompanied by changes of mitochondrial structure. Although this led to the release of a large number of cytochrome c, but did not induce programmed apoptosis. Moreover, GSEA enrichment analysis reveals that metabolic regulation may be the main mechanism of IMMT involved in BC progression, and the key indexes of glycolysis are also obviously inhibited after IMMT-KD. Collectively, these findings support that IMMT maybe a promising new target for metabolic therapy in BC.

## Supplementary Information

Supplementary Information 1.

Supplementary Information 2.

Supplementary Information 3.

Supplementary Table S1.

Supplementary Table S2.

Supplementary Table S3.

Supplementary Table S4.

## Data Availability

Public data were shared and available on the following websites: TCGA (http://cancergenome.nih.gov/), The K-M Plotter Tool (http://kmplot.com/analysis/), HPA online tool (http://www.proteatlas.org), GEPIA (https://GEPIA.cancer-PKU.cn/) and TIMER2.0 (http://timer.cistrome.org/). Most of the results of the current study appear in the article or as supplementary materials. In case of reasonable request, the corresponding author can provide further details regarding the data. Generated Statement: The raw data supporting the conclusions of this article will be made available by the authors, without undue reservation.
