# Author Correction: Suppressing mitochondrial inner membrane protein (IMMT) inhibits the proliferation of breast cancer cells through mitochondrial remodeling and metabolic regulation

**DOI:** 10.1038/s41598-024-74030-2

**Published:** 2024-10-25

**Authors:** Li Liu, Qingqing Zhao, Daigang Xiong, Dan Li, Jie Du, Yunfei Huang, Yan Yang, Rui Chen

**Affiliations:** 1 Clinical Medical College, Zunyi Medical University, Zunyi, China; 2 Department of Laboratory Medicine, Affiliated Hospital of ZunYi Medical University, Zunyi, China; 3 School of Laboratory Medicine, Zunyi Medical University, Zunyi, China; 4 Department of General Surgery, Affiliated Hospital of Zunyi Medical University, Zunyi, China; 5 Department of Thyroid and Breast Surgery, Affiliated Hospital of Zunyi Medical University, Zunyi, China

Correction to: *Scientific Reports* 10.1038/s41598-024-63427-8, published online 04 June 2024

The original version of this Article contained an error in Figure 3, panel E, where two cloned formation images were duplicated. The original Figure 3 and accompanying legend appear below.

**Fig. 3:**
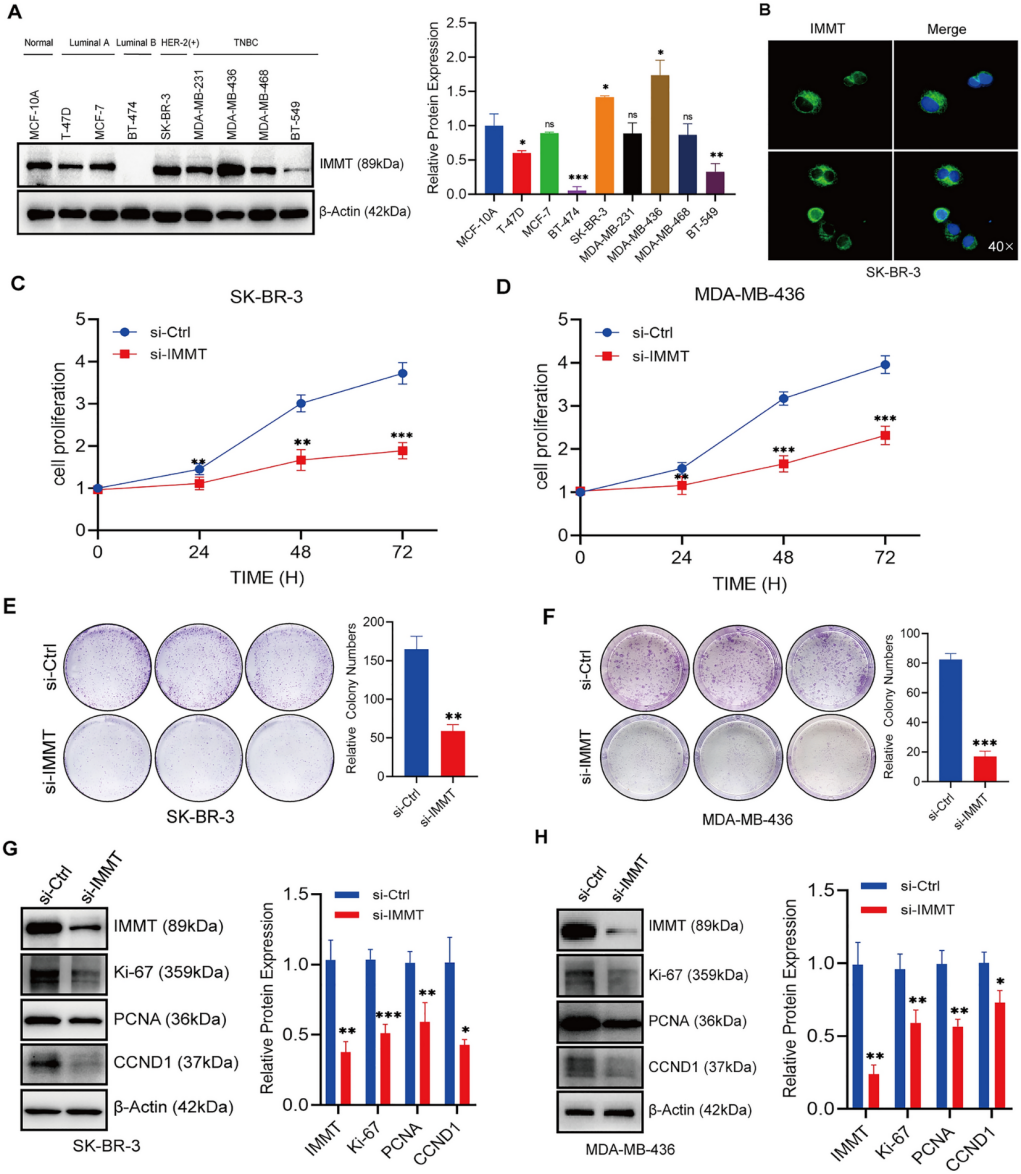
The proliferation of BC cells is inhibited in the absence of IMMT. (**A**) This is supported by the expression of IMMT protein in various types of BC cell lines. Mean ± SD (n = 3). (**B**) The immunofluorescence localization of IMMT in SK-BR-3 cells. (**C**, **D**) CCK-8 assays revealed suppressed proliferation in SK-BR-3 and MDA-MB-436 cells upon knockdown of IMMT. Mean ± SD (n = 3 to 4). (**E**, **F**) The decrease in colony counts of SK-BR-3 and MDA-MB-436 cells transfected with si-IMMT compared to the si-Ctrl group. (**G**, **H**) This was confirmed through Western blot analysis of proliferation-related proteins following si-IMMT transfection. Data are presented as mean ± SEM, *P < 0.05, **P < 0.01 and ***P < 0.001.

The original Article has been corrected.

