# Supplementary figures and images for: Suppressing mitochondrial inner membrane protein (IMMT) inhibits the proliferation of breast cancer cells through mitochondrial remodeling and metabolic regulation
Source: Sci Rep. 2024 Jun 4;14:12766. doi: 10.1038/s41598-024-63427-8 (PMC11150385; doi:10.1038/s41598-024-63427-8)

# Hazard ratio

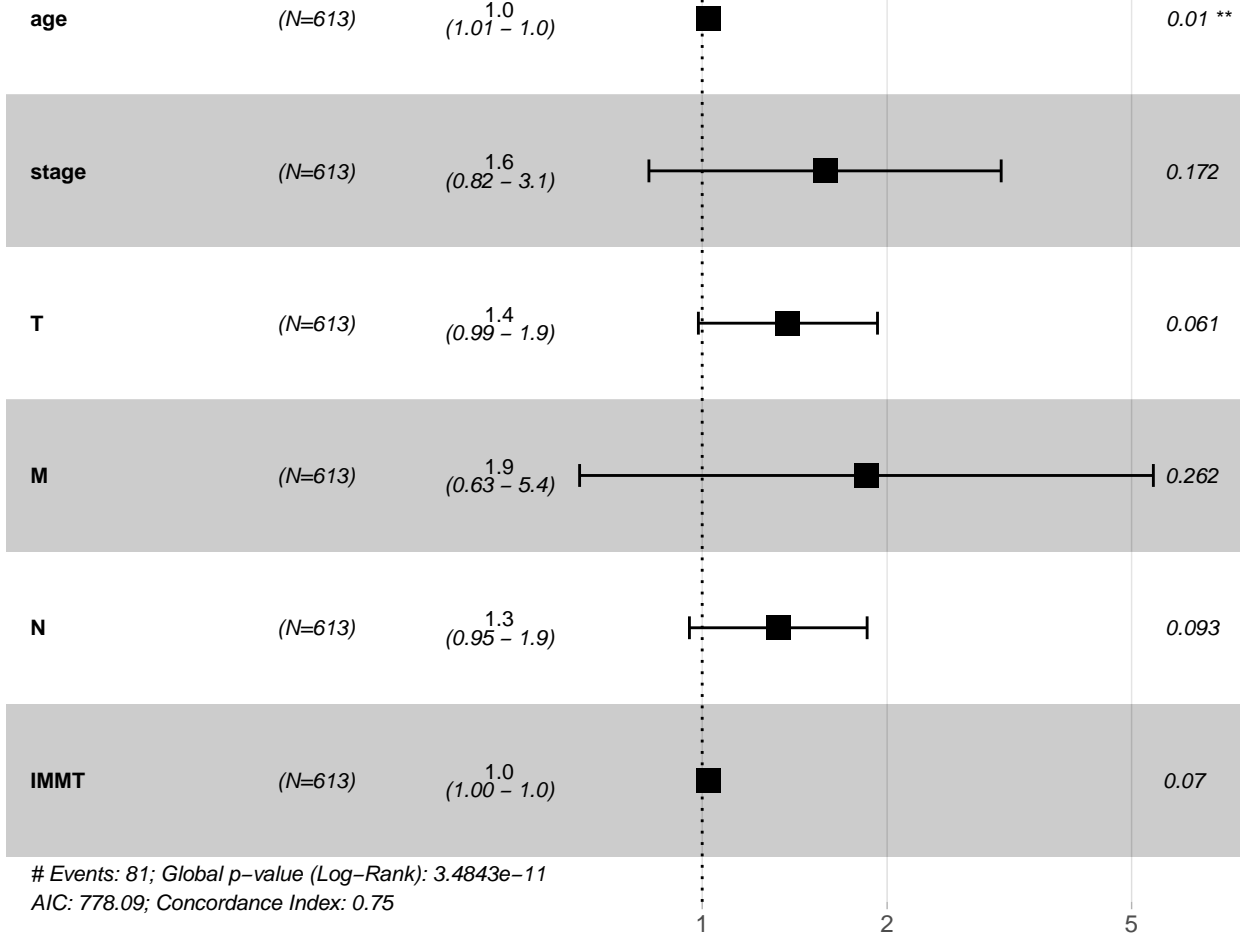

Supplement: Supplementary file 1 — Supplementary Information 1. [file 41598_2024_63427_MOESM1_ESM.pdf]

# Hazard ratio

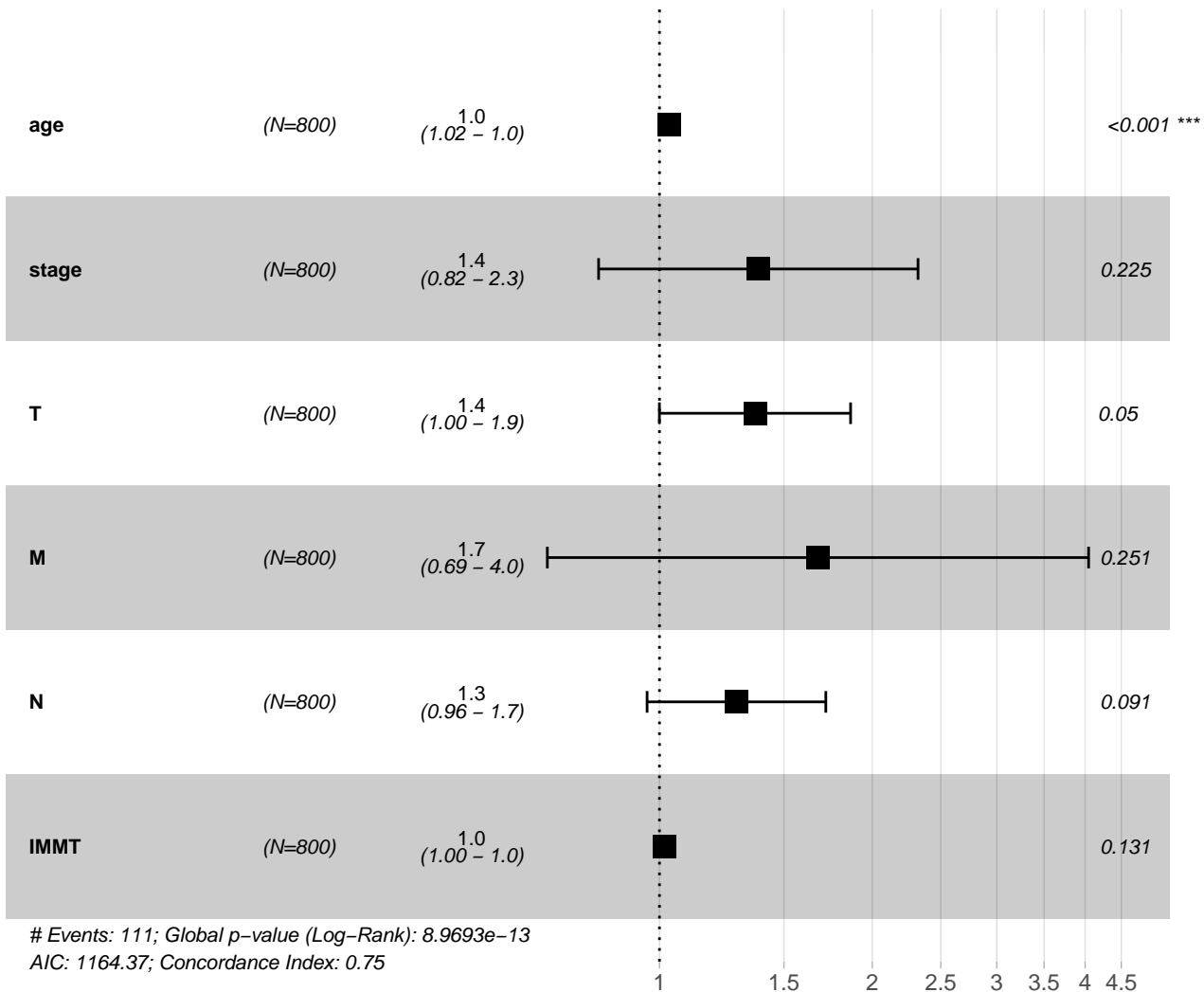

Supplement: Supplementary file 2 — Supplementary Information 2. [file 41598_2024_63427_MOESM2_ESM.pdf]
