# Supplementary material for: Suppressing mitochondrial inner membrane protein (IMMT) inhibits the proliferation of breast cancer cells through mitochondrial remodeling and metabolic regulation
Source: Sci Rep. 2024 Jun 4;14:12766. doi: 10.1038/s41598-024-63427-8 (PMC11150385; doi:10.1038/s41598-024-63427-8)
Supplement: Supplementary file 3 — Supplementary Information 3. [file 41598_2024_63427_MOESM3_ESM.pdf]

Figure 3A

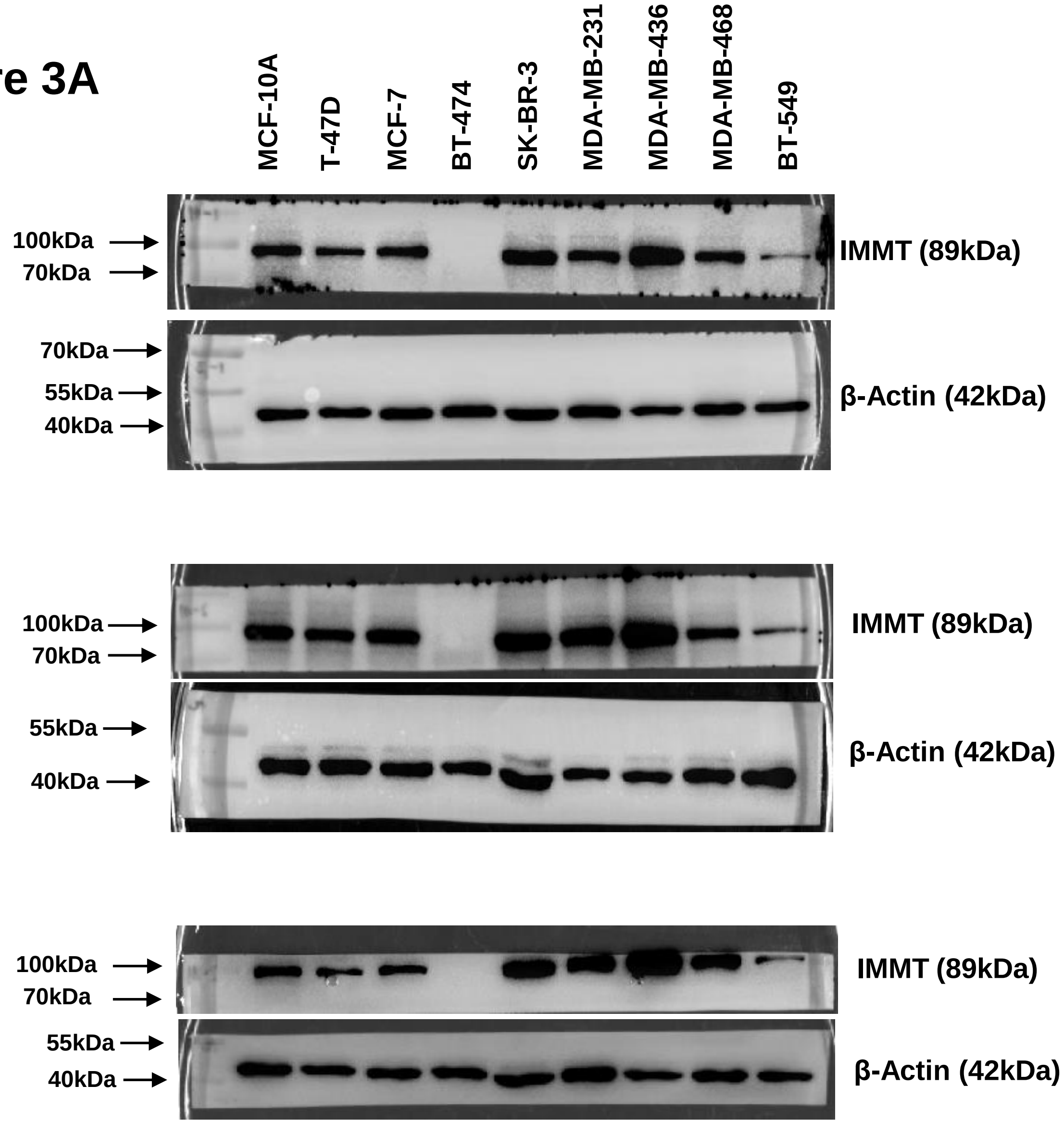
MCF-10A
T-47D
MCF-7
BT-474
SK-BR-3
MDA-MB-231
MDA-MB-436
MDA-MB-468
BT-549
100kDa
70kDa
IMMT (89kDa)
70kDa
55kDa
40kDa
β-Actin (42kDa)
100kDa
70kDa
IMMT (89kDa)
55kDa
40kDa
β-Actin (42kDa)
100kDa
70kDa
IMMT (89kDa)
55kDa
40kDa
β-Actin (42kDa)

Figure 3G

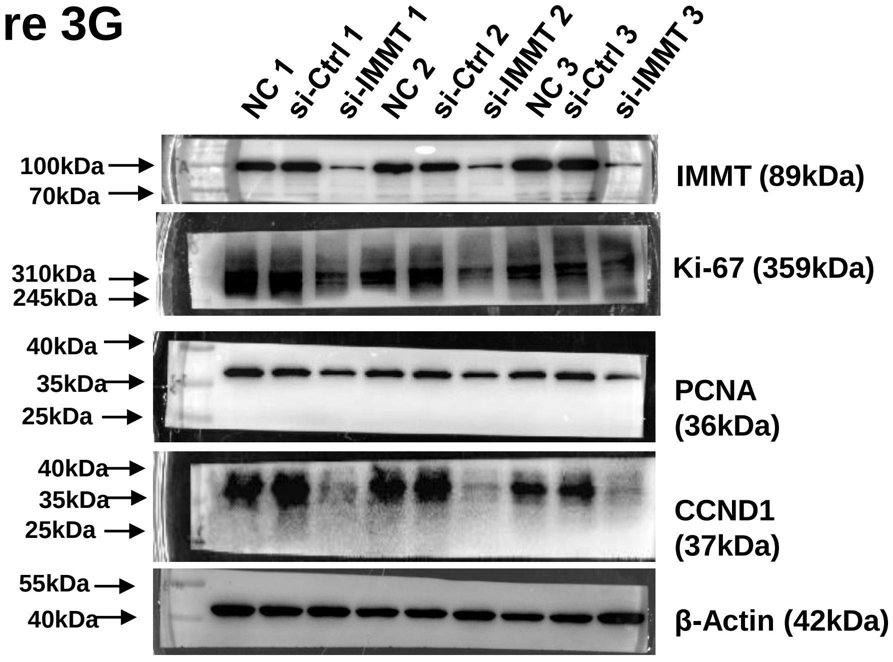

SK-BR-3

Figure 3H

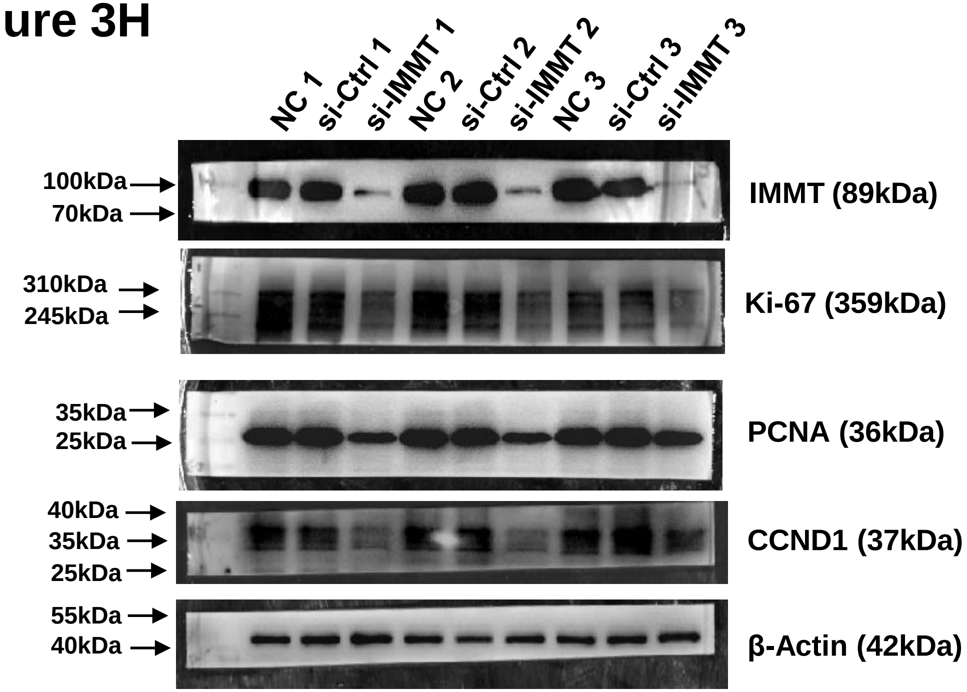

MDA-MB-436

Figure 4B

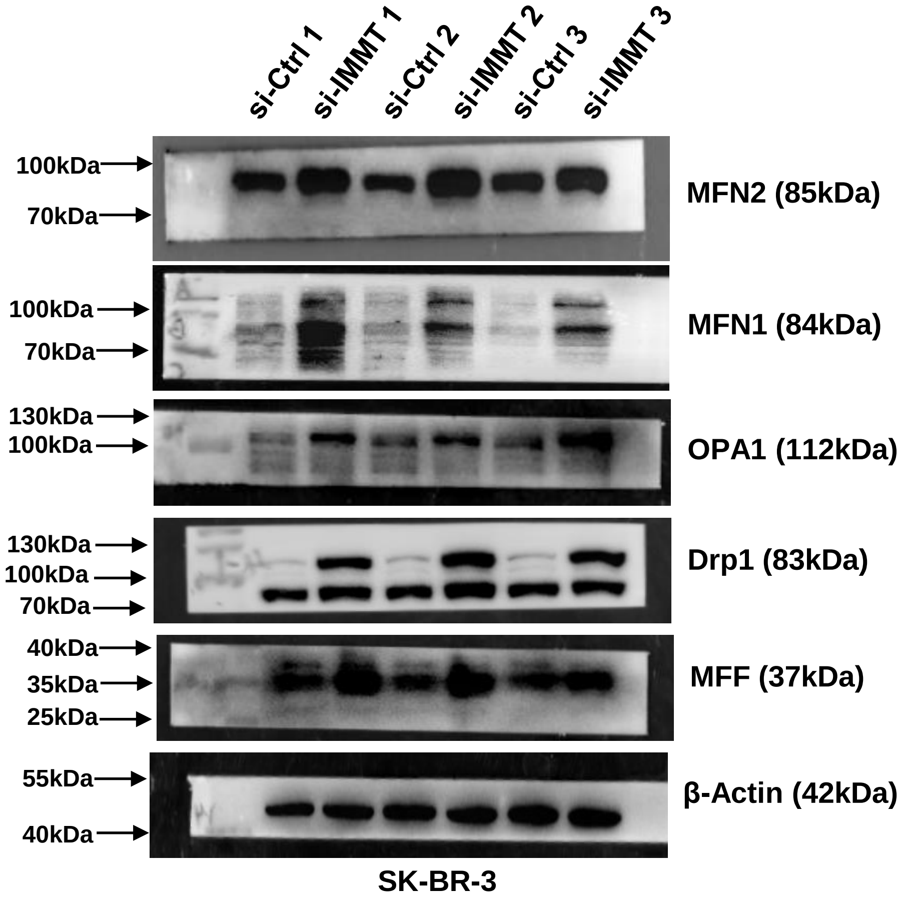
si-Ctrl 1
si-IMMT 1
si-Ctrl 2
si-IMMT 2
si-Ctrl 3
si-IMMT 3
100kDa
70kDa
MFN2 (85kDa)
100kDa
70kDa
MFN1 (84kDa)
130kDa
100kDa
OPA1 (112kDa)
130kDa
100kDa
70kDa
Drp1 (83kDa)
40kDa
35kDa
25kDa
MFF (37kDa)
55kDa
40kDa
β-Actin (42kDa)
SK-BR-3

Figure 5B

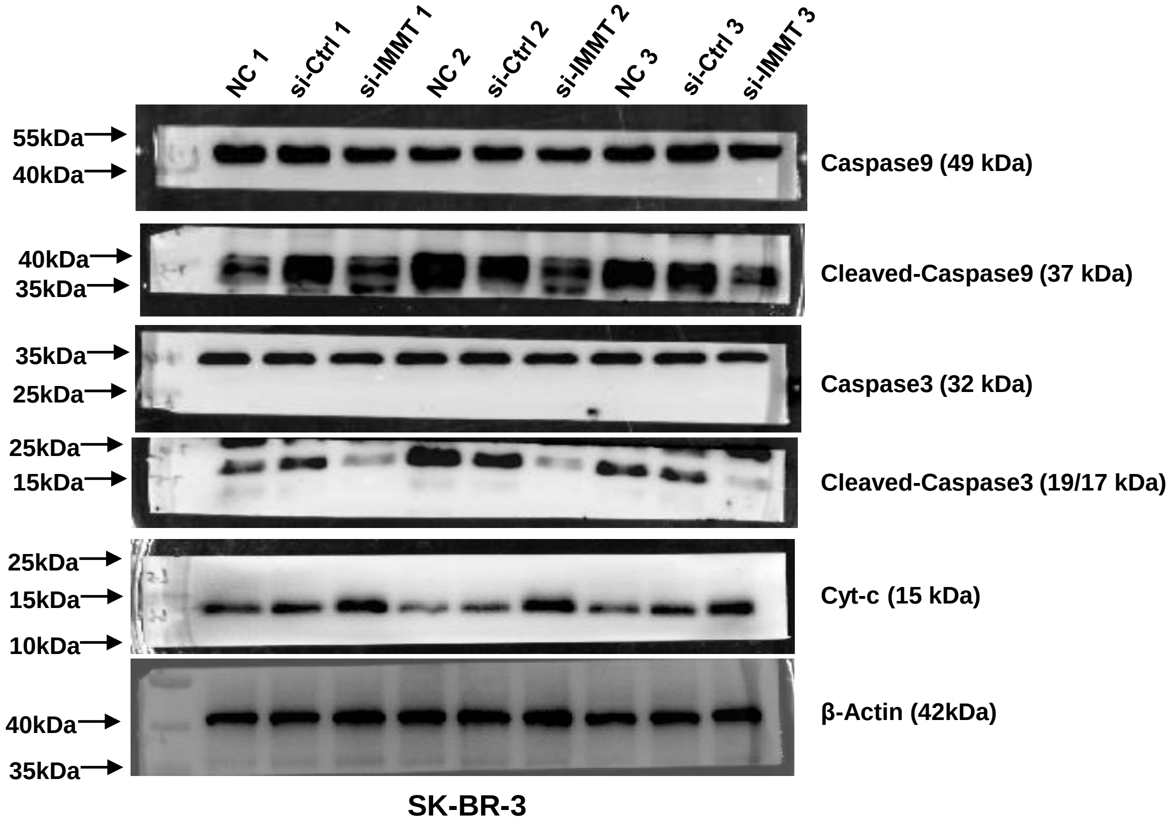
NC 1
si-Ctrl 1
si-IMMT 1
NC 2
si-Ctrl 2
si-IMMT 2
NC 3
si-Ctrl 3
si-IMMT 3
55kDa
40kDa
Caspase9 (49 kDa)
40kDa
35kDa
Cleaved-Caspase9 (37 kDa)
35kDa
25kDa
Caspase3 (32 kDa)
25kDa
15kDa
Cleaved-Caspase3 (19/17 kDa)
25kDa
15kDa
10kDa
Cyt-c (15 kDa)
40kDa
35kDa
β-Actin (42kDa)
SK-BR-3

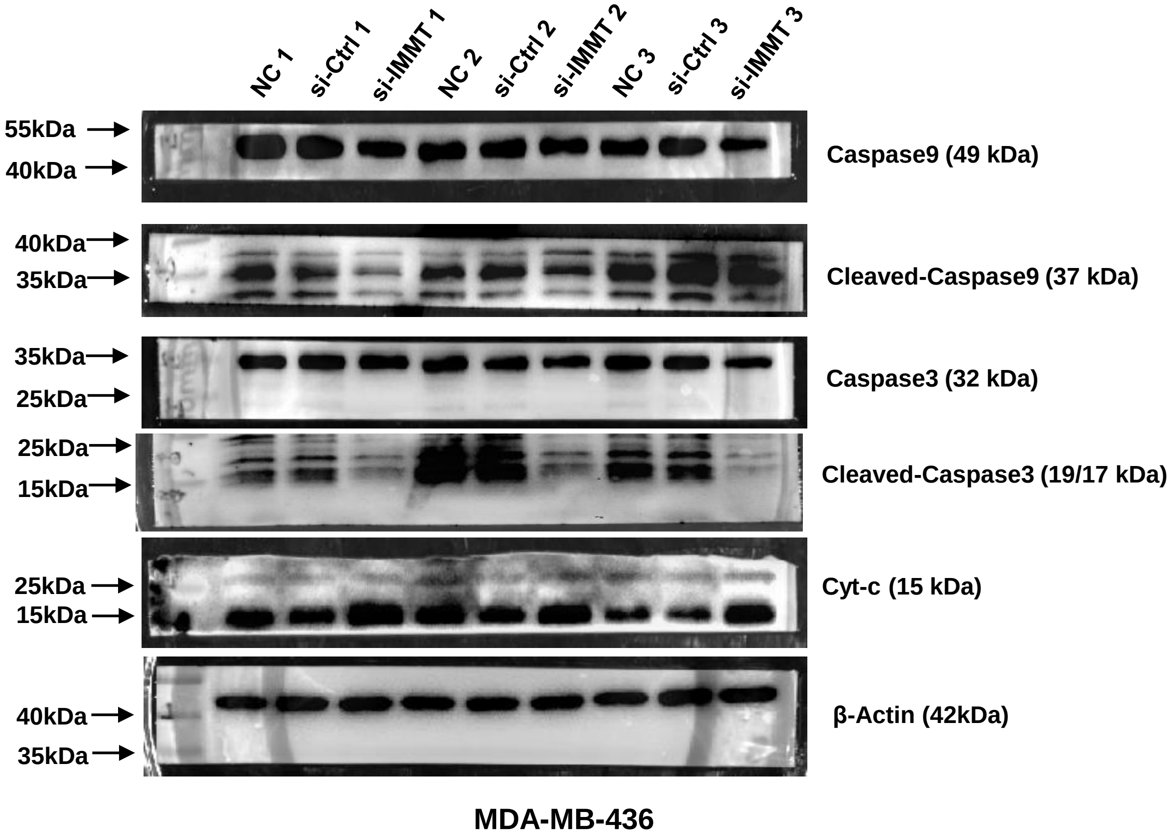
NC 1
si-Ctrl 1
si-IMMT 1
NC 2
si-Ctrl 2
si-IMMT 2
NC 3
si-Ctrl 3
si-IMMT 3
55kDa
40kDa
Caspase9 (49 kDa)
40kDa
35kDa
Cleaved-Caspase9 (37 kDa)
35kDa
25kDa
Caspase3 (32 kDa)
25kDa
15kDa
Cleaved-Caspase3 (19/17 kDa)
25kDa
15kDa
Cyt-c (15 kDa)
40kDa
35kDa
β-Actin (42kDa)
MDA-MB-436

Figure 6E

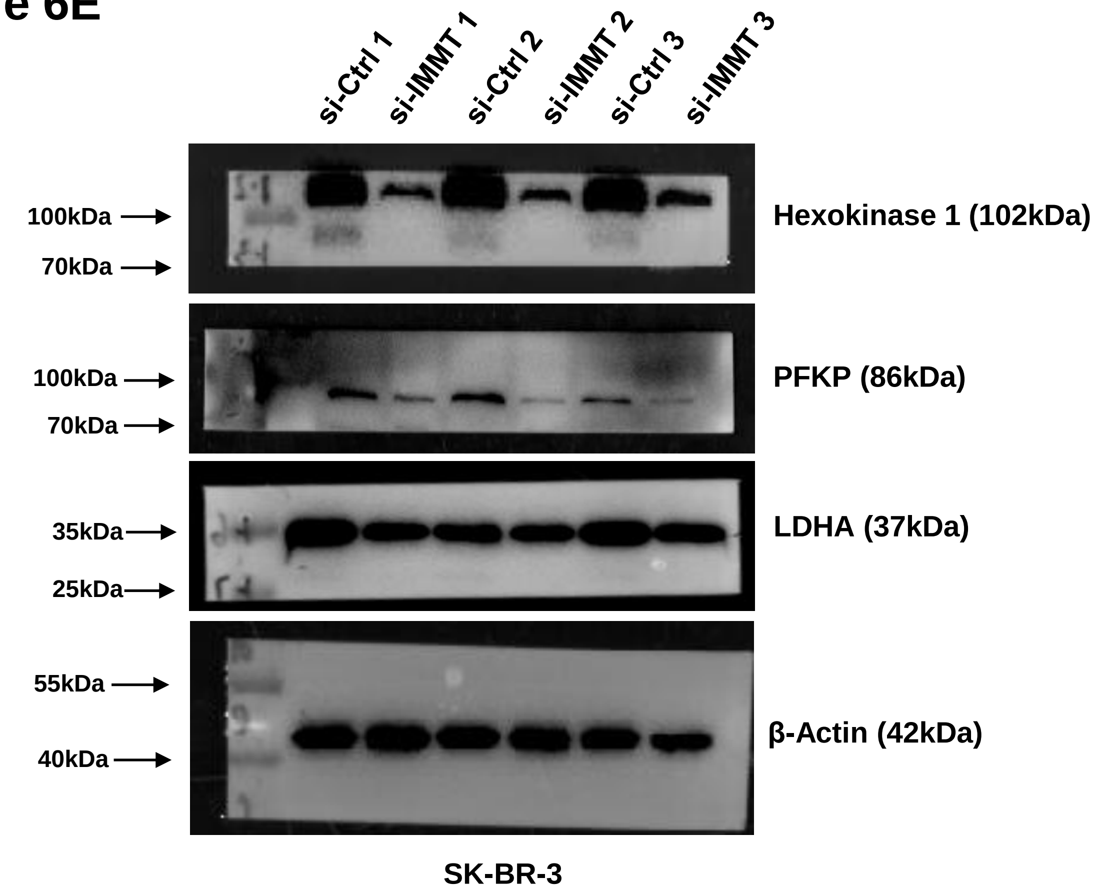
si-Ctrl 1
si-IMMT 1
si-Ctrl 2
si-IMMT 2
si-Ctrl 3
si-IMMT 3
100kDa
70kDa
Hexokinase 1 (102kDa)
100kDa
70kDa
PFKP (86kDa)
35kDa
25kDa
LDHA (37kDa)
55kDa
40kDa
β-Actin (42kDa)
SK-BR-3
