# Supplementary material for: Suppressing mitochondrial inner membrane protein (IMMT) inhibits the proliferation of breast cancer cells through mitochondrial remodeling and metabolic regulation
Source: Sci Rep. 2024 Jun 4;14:12766. doi: 10.1038/s41598-024-63427-8 (PMC11150385; doi:10.1038/s41598-024-63427-8)
Supplement: Supplementary file 4 — Supplementary Table S1. [file 41598_2024_63427_MOESM4_ESM.docx]

**Table S1.** Cox regression univariate and multivariate analysis of various prognostic parameters in BC patients based on TCGA database.

|  | Univariate analysis | | |  | Multivariate analysis | | |
| --- | --- | --- | --- | --- | --- | --- | --- |
|  | P | Hazard | 95% confidence |  | P | Hazard | 95% confidence |
|  |  | Ratio | interval |  |  | Ratio | interval |
| Age | 1.34E-05 | 1.033 | 1.018-1.048 |  | 7.69E-06 | 1.035 | 1.019-1.050 |
| Stage | 1.16E-09 | 2.089 | 1.648-2.649 |  | 0.1036 | 1.545 | 0.915-2.610 |
| T | 0.0002 | 1.511 | 1.213-1.882 |  | 0.8832 | 1.023 | 0.754-1.389 |
| M | 4.58E-09 | 6.016 | 3.302-10.963 |  | 0.3118 | 1.552 | 0.662-3.635 |
| N | 6.40E-08 | 1.681 | 1.393-2.030 |  | 0.1298 | 1.259 | 0.935-1.696 |
| IMMT | 0.0551 | 1.019 | 0.999-1.040 |  | 0.0303 | 1.022 | 1.002-1.042 |
