# Supplementary material for: Suppressing mitochondrial inner membrane protein (IMMT) inhibits the proliferation of breast cancer cells through mitochondrial remodeling and metabolic regulation
Source: Sci Rep. 2024 Jun 4;14:12766. doi: 10.1038/s41598-024-63427-8 (PMC11150385; doi:10.1038/s41598-024-63427-8)
Supplement: Supplementary file 5 — Supplementary Table S2. [file 41598_2024_63427_MOESM5_ESM.docx]

**Table S2.** Enrichment analysis of metabolic pathway based on differential expression of IMMT in TCGA database.

| NAME NES (normalize enrichment score) NOM p-val FDR q-val | | | |
| --- | --- | --- | --- |
| KEGG_PYRIMIDINE_METABOLISM | 2.5280097 | 0 | 0 |
| KEGG_PURINE_METABOLISM | 2.4038513 | 0 | 0 |
| KEGG_CYSTEINE_AND_METHIONINE_METABOLISM | 2.2770052 | 0 | 3.09E-04 |
| KEGG_PYRUVATE_METABOLISM | 2.2730381 | 0 | 2.93E-04 |
| KEGG_FRUCTOSE_AND_MANNOSE_METABOLISM | 2.2153988 | 0 | 3.67E-04 |
| KEGG_ALANINE_ASPARTATE_AND_GLUTAMATE_METABOLISM | 2.2109518 | 0 | 3.52E-04 |
| KEGG_PROPANOATE_METABOLISM | 2.199629 | 0 | 4.31E-04 |
| KEGG_BUTANOATE_METABOLISM | 2.1977656 | 0 | 4.15E-04 |
| KEGG_GLYOXYLATE_AND_DICARBOXYLATE_METABOLISM | 2.179483 | 0 | 6.27E-04 |
| KEGG_GALACTOSE_METABOLISM | 2.15032 | 0 | 9.09E-04 |
| KEGG_TRYPTOPHAN_METABOLISM | 2.084077 | 0 | 0.001922028 |
| KEGG_AMINO_SUGAR_AND_NUCLEOTIDE_SUGAR_METABOLISM | 2.0792885 | 0.001919386 | 0.001868126 |
| KEGG_SELENOAMINO_ACID_METABOLISM | 1.9917278 | 0.003913894 | 0.005096867 |
| KEGG_FATTY_ACID_METABOLISM | 1.8728807 | 0.003891051 | 0.012630846 |
| KEGG_ARGININE_AND_PROLINE_METABOLISM | 1.850996 | 0.003960396 | 0.015061664 |
| KEGG_BETA_ALANINE_METABOLISM | 1.7805953 | 0.013513514 | 0.02430229 |
| KEGG_GLUTATHIONE_METABOLISM | 1.7572396 | 0.005769231 | 0.026718153 |
| KEGG_INOSITOL_PHOSPHATE_METABOLISM | 1.6669192 | 0.027888447 | 0.043995705 |
| KEGG_PORPHYRIN_AND_CHLOROPHYLL_METABOLISM | 1.6462452 | 0.025490196 | 0.04827221 |
