# Supplementary material for: Suppressing mitochondrial inner membrane protein (IMMT) inhibits the proliferation of breast cancer cells through mitochondrial remodeling and metabolic regulation
Source: Sci Rep. 2024 Jun 4;14:12766. doi: 10.1038/s41598-024-63427-8 (PMC11150385; doi:10.1038/s41598-024-63427-8)
Supplement: Supplementary file 6 — Supplementary Table S3. [file 41598_2024_63427_MOESM6_ESM.docx]

**Table S3.** Co-expression values and binding scores of 16 factors with which the STRING web analytics IMMT interacts.

| Node1 | Node2 | Co-expression | Combined score |
| --- | --- | --- | --- |
| IMMT | DLD | 0.303 | 0.479 |
| IMMT | ACO2 | 0.184 | 0.514 |
| IMMT | PIK3CA | 0 | 0.428 |
| IMMT | DGUOK | 0.066 | 0.52 |
| IMMT | PDE3B | 0 | 0.472 |
| IMMT | PIK3CB | 0 | 0.563 |
| IMMT | PDHB | 0.246 | 0.479 |
| IMMT | PIK3CG | 0 | 0.977 |
| IMMT | SGPL1 | 0.053 | 0.74 |
| IMMT | PIK3CD | 0 | 0.576 |
| IMMT | SUCLA2 | 0.28 | 0.679 |
| IMMT | PDHA1 | 0.196 | 0.566 |
| IMMT | SUCLG1 | 0.285 | 0.521 |
| IMMT | AK4 | 0.084 | 0.686 |
| IMMT | PAFAH1B1 | 0 | 0.483 |
| IMMT | FBP1 | 0 | 0.439 |
