# Supplementary material for: Suppressing mitochondrial inner membrane protein (IMMT) inhibits the proliferation of breast cancer cells through mitochondrial remodeling and metabolic regulation
Source: Sci Rep. 2024 Jun 4;14:12766. doi: 10.1038/s41598-024-63427-8 (PMC11150385; doi:10.1038/s41598-024-63427-8)
Supplement: Supplementary file 7 — Supplementary Table S4. [file 41598_2024_63427_MOESM7_ESM.docx]

**Table S4.** Types of interactions between IMMT and interaction complexes.

| IMMT-ACO2 |  |  |  |  |
| --- | --- | --- | --- | --- |
| CHAIN A | Residue | CHAIN B | Residue | Interaction type |
| IMMT | Y656 | ACO2 | D139 | Hbond |
| IMMT | Q657 | ACO2 | K605 | Hbond |
| IMMT | Y656 | ACO2 | E131 | Hbond |
| IMMT | Y662 | ACO2 | K462 | Hbond |
| IMMT | N686 | ACO2 | D645 | Hbond |
| IMMT | K675 | ACO2 | E639 | Hbond |
| IMMT | Q672 | ACO2 | K628 | Hbond |
| IMMT | F669 | ACO3 | N630 | Hbond |
| IMMT | E683 | ACO4 | K652 | Salt bridge |

| IMMT-PAFAH1B1 |  |  |  |  |
| --- | --- | --- | --- | --- |
| CHAIN A | Residue | CHAIN B | Residue | Interaction type |
| IMMT | L666 | PAFAH1B1 | Q154 | Hbond |
| IMMT | Y662 | PAFAH1B1 | V111 | Hbond |
| IMMT | R644 | PAFAH1B1 | P110 | Hbond |
| IMMT | R644 | PAFAH1B1 | S127 | Hbond |
| IMMT | K640 | PAFAH1B1 | D129 | Hbond |
| IMMT | Y662 | PAFAH1B1 | E128 | Hbond |

IMMT-PDHA1

| CHAIN A | Residue | CHAIN B | Residue | Interaction type |
| --- | --- | --- | --- | --- |
| IMMT | R644 | PDHA1 | S341 | Hbond |
| IMMT | N686 | PDHA1 | Q174 | Hbond |
| IMMT | Q672 | PDHA1 | N155 | Hbond |

IMMT-DGUOK

| CHAIN A | Residue | CHAIN B | Residue | Interaction type |
| --- | --- | --- | --- | --- |
| IMMT | D723 | DGUOK | R105 | Hbond |
| IMMT | R644 | DGUOK | T112 | Hbond |
| IMMT | Y662 | DGUOK | F115 | Hbond |
| IMMT | L666 | DGUOK | K120 | Hbond |

| IMMT-PIK3CA |  |  |  |  |
| --- | --- | --- | --- | --- |
| CHAIN A | Residue | CHAIN B | Residue | Interaction type |
| IMMT | P618 | PIK3CA | H1060 | Hbond |
| IMMT | D723 | PIK3CA | R949 | Hbond |
| IMMT | R644 | PIK3CA | E453 | Salt bridge |
| IMMT | Y662 | PIK3CA | L452 | Hbond |

IMMT-PIK3CB

| CHAIN A | Residue | CHAIN B | Residue | Interaction type |
| --- | --- | --- | --- | --- |
| IMMT | Y662 | PIK3CB | L15 | Hbond |
| IMMT | V720 | PIK3CB | Q43 | Hbond |
